# Identification of novel leishmanicidal molecules by virtual and biochemical screenings targeting *Leishmania* eukaryotic translation initiation factor 4A

**DOI:** 10.1371/journal.pntd.0006160

**Published:** 2018-01-18

**Authors:** Emna Harigua-Souiai, Yosser Zina Abdelkrim, Imen Bassoumi-Jamoussi, Ons Zakraoui, Guillaume Bouvier, Khadija Essafi-Benkhadir, Josette Banroques, Nathan Desdouits, Hélène Munier-Lehmann, Mourad Barhoumi, N. Kyle Tanner, Michael Nilges, Arnaud Blondel, Ikram Guizani

**Affiliations:** 1 Laboratory of Molecular Epidemiology and Experimental Pathology – LR11IPT04, Institut Pasteur de Tunis, Université de Tunis el Manar, Tunis, Tunisia; 2 Institut Pasteur, Unité de Bioinformatique Structurale, CNRS UMR 3528, Département de Biologie Structurale et Chimie, Paris, France; 3 Laboratory of Microbial Gene Expression (EGM), CNRS UMR8261/Université Paris Diderot P7, Sorbonne Paris Cité & PSL, Institut de Biologie Physico-Chimique, Paris, France; 4 Faculté des Sciences de Bizerte, Université de Carthage, Tunis, Tunisia; 5 Institut Pasteur, Unité de Chimie et Biocatalyse, Département de Biologie Structurale et Chimie, Paris, France; 6 Unité Mixte de Recherche 3523, Centre National de la Recherche Scientifique, Paris, France; National Institutes of Health, UNITED STATES

## Abstract

Leishmaniases are neglected parasitic diseases in spite of the major burden they inflict on public health. The identification of novel drugs and targets constitutes a research priority. For that purpose we used *Leishmania infantum* initiation factor 4A (LieIF), an essential translation initiation factor that belongs to the DEAD-box proteins family, as a potential drug target. We modeled its structure and identified two potential binding sites. A virtual screening of a diverse chemical library was performed for both sites. The results were analyzed with an in-house version of the Self-Organizing Maps algorithm combined with multiple filters, which led to the selection of 305 molecules. Effects of these molecules on the ATPase activity of LieIF permitted the identification of a promising hit (**208**) having a half maximal inhibitory concentration (IC_50_) of 150 ± 15 *μ*M for 1 *μ*M of protein. Ten chemical analogues of compound **208** were identified and two additional inhibitors were selected (**20** and **48**). These compounds inhibited the mammalian eIF4I with IC_50_ values within the same range. All three hits affected the viability of the extra-cellular form of *L. infantum* parasites with IC_50_ values at low micromolar concentrations. These molecules showed non-significant toxicity toward THP-1 macrophages. Furthermore, their anti-leishmanial activity was validated with experimental assays on *L. infantum* intramacrophage amastigotes showing IC_50_ values lower than 4.2 *μ*M. Selected compounds exhibited selectivity indexes between 19 to 38, which reflects their potential as promising anti-*Leishmania* molecules.

## Author summary

Leishmaniases constitute a group of neglected parasitic diseases that inflict major burden on public health. Novel drugs and targets need to be identified since current therapies have adverse side effects. Herein, we focused on *Leishmania infantum* translation initiation factor 4A (LieIF), as a potential drug target. LieIF, a pivotal enzyme in the translation machinery, is also implicated in host-pathogen interactions. We modeled its 3D structure and identified two pockets, which were used in virtual screenings of a chemical compound library. Therefore, we selected and purchased 305 compounds. We established a reliable ATPase screening assay to test the molecules against the enzymatic activity of LieIF and its mammalian homologue. A promising hit was retained and further characterized. It inhibited both proteins but showed different kinetic properties. It was used as a basis to identify similar analogues and two additional inhibitors were identified. All three hits reduced the viability of the extracellular promastigote form of the parasite, but they had no significant cytotoxic effects on host cells. They also affected the viability of the intracellular amastigote form and reduced the macrophage infection. This selectivity is very promising and indicates that these inhibitors would constitute an avenue to develop strategies to fight leishmaniases.

## Introduction

Leishmaniases are neglected diseases caused by multiple protozoan parasite species of the genus *Leishmania*. These vector-borne diseases affect more than 98 countries that are mainly developing countries with limited public health resources. Additionally, more than 300 million people are at risk of being infected. [1] Three different clinical forms are described: Cutaneous Leishmaniasis (CL), Muco-Cutaneous Leishmaniasis (MCL) and the most severe Visceral Leishmaniasis (VL), which is fatal if left untreated. Each year, 1.5 to 2 million cases are reported, among which 0.5 million are cases of VL that cause 40,000 deaths per year. [1]

Currently available control measures are mainly based on diagnosis, patient treatment and vector control. Mainstay therapy is based on the use of pentavalent antimonials. [2, 3] Commonly used second-line drugs are miltefosine, amphotericin B, liposomal amphotericin B and paromomycin. All these treatments are given by injections, except for miltefosine that is administered orally. [4] They require long treatment courses, are toxic and costly, and have adverse effects. [3, 5, 6] The identification of novel drug targets, therapeutic molecules or immune modulators that enhance the response to treatment constitute research priorities, particularly against the fatal VL mainly caused by *L. donovani* and *L. infantum*. [7] Different criteria help to define potential drug targets: these include expression in relevant life stages, unique genetic or biochemical properties including essentiality, druggability and structural features that allow selection of inhibitors, and assayability. [8] Different targets are being investigated. [8] We focus our work on the *Leishmania infantum* translation initiation factor 4A (LieIF). [9]

Translation factors play key roles in the cell and they are considered as relevant drug targets in cancers. In particular, the translation initiation factor eIF4A, [10–14] the prototype of the DEAD box proteins (DBPs) family, is considered a potent target. [15, 16] It plays a pivotal role in the translation initiation complex eIF4F as an essential enzyme. [17, 18] In *Leishmania infantum*, experimental evidence assigned an eIF4A-like functional role to a protein called LieIF that is encoded by *LinJ.01.0790/LinJ.01.0800* genes mapping to chromosome 1 (*LmjF.01.0770/LmjF.01.0780* in *L. major*). [9, 19, 20] In previous investigations, we have shown that LieIF has the ability to bind to eIF4G of yeast *in vitro*, and that it has a dominant-negative phenotype when expressed in yeast, resulting in growth reduction. [9] LieIF is expressed in both parasite stages [21–25], and it is involved in kinases signaling cascades of the amastigotes leading to its phosphorylation on THR135. [25] Interestingly, it has been identified among proteins of the secretome and exosomes of infectious promastigotes of *L. infantum* [26, 27] and of *L. donovani*. [28] In addition, LieIF is a vaccine subunit that exerts a natural Th1-type adjuvant property. [29, 30]

**Fig 1:**
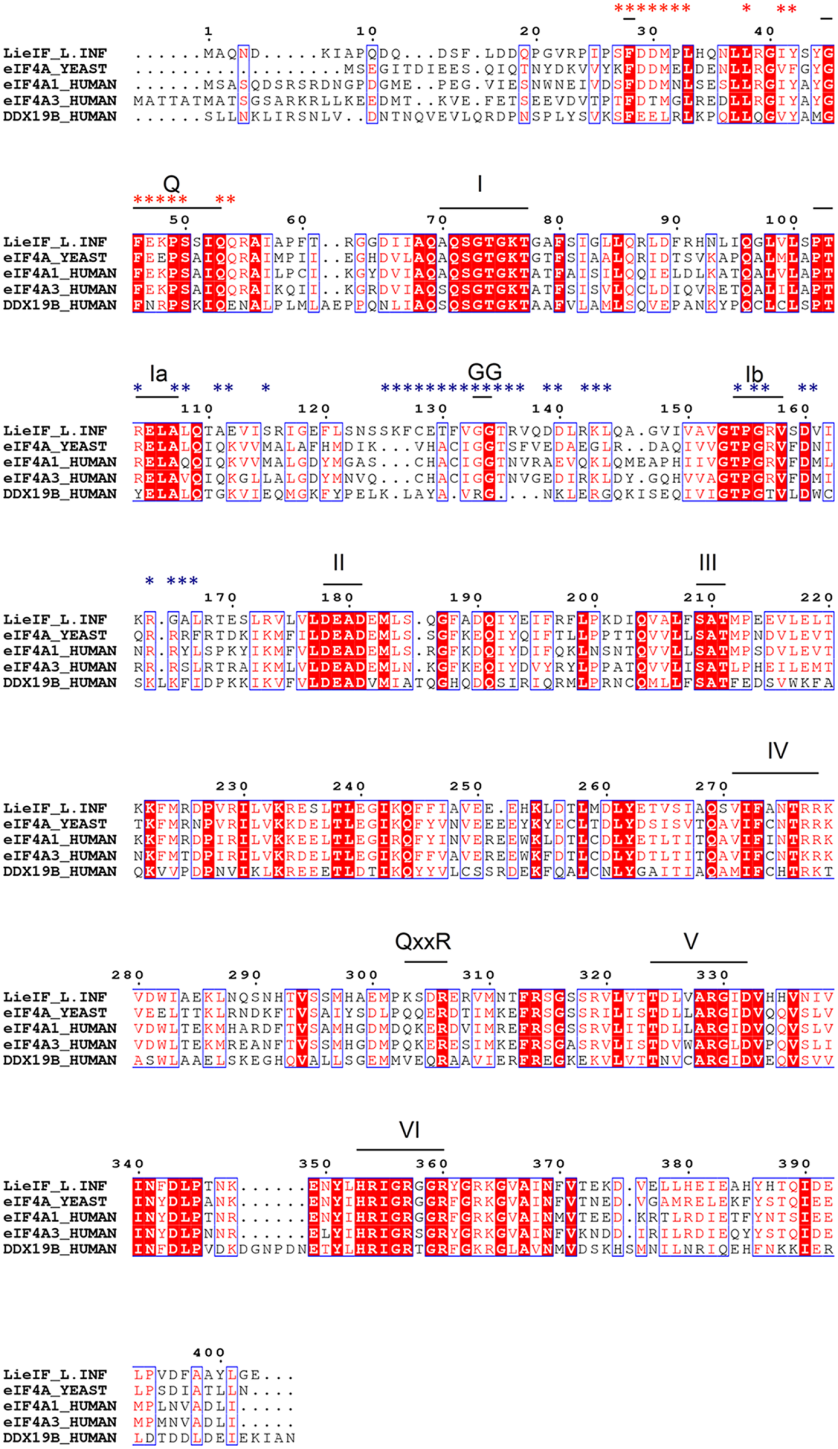
Alignment of LieIF with its homologs eIF4AI_hu_ (DDX2A), eIF4AIII_hu_ (DDX48), eIF4A_yeast_ and a more distantly related DBP involved in RNA transport DDX19B_hu_ (Δ:1-60). Residues constituting pocket P1 are marked with red stars and those constituting P2 are marked with blue stars. Conserved motifs are indicated on top of the LieIF sequence. Domain 1 contains motifs Q [F–FxxPTxIQ], I [AxxGxGKT], Ia [PTRELA], GG [GG], Ib [TPGRx], II [DEAD] and III [SAT]. Domain 2, contains motifs IV [IIFxpppp] (where “p” denotes polar/charged residues), QxxR, V [TDxxARGxD] and VI [HRxGRxxR].

**Fig 2:**
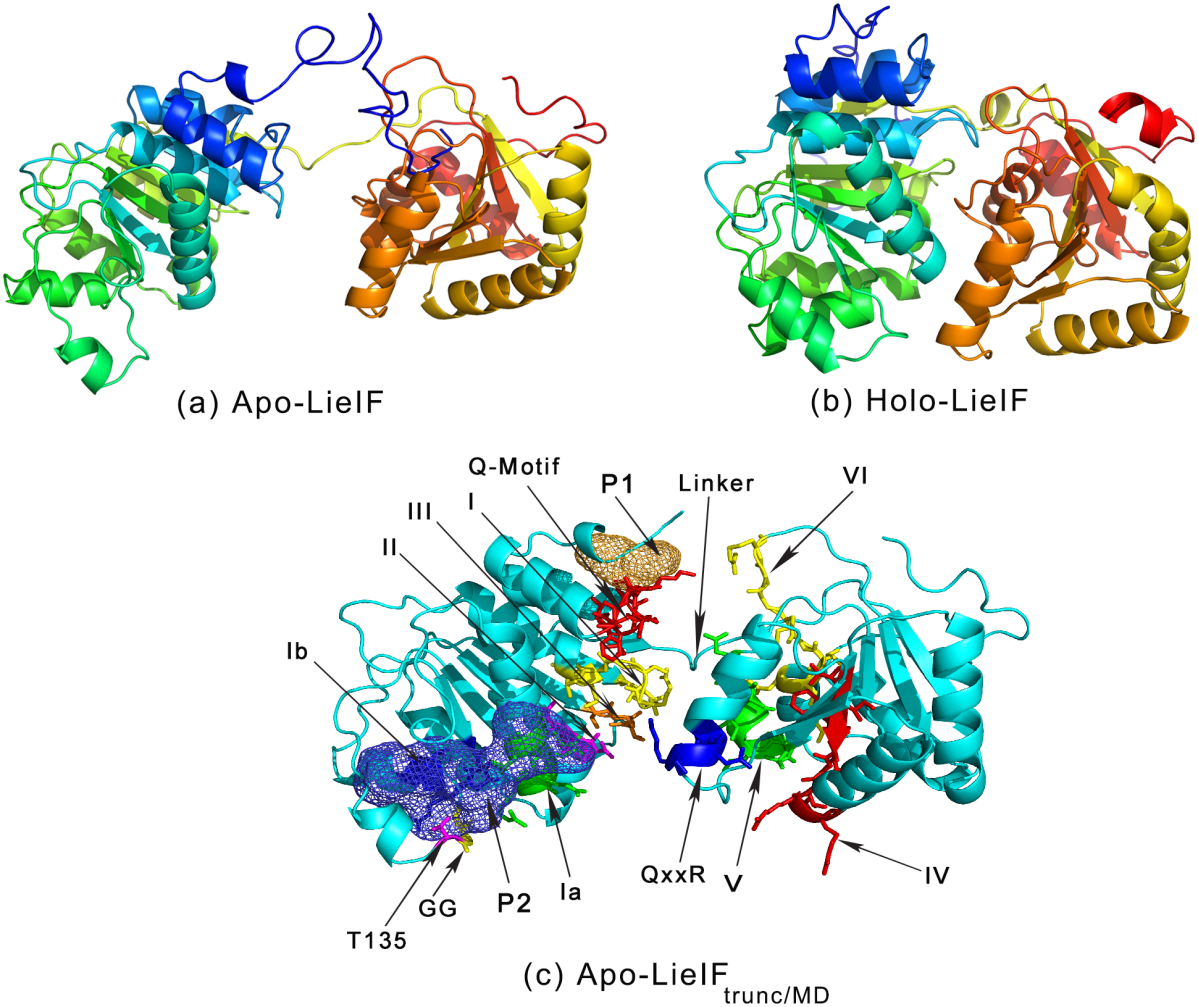
LieIF models and pockets. (a) Apo-LieIF corresponds to the open conformation of LieIF and (b) Holo-LieIF corresponds to its closed conformation. The blue to red color gradient goes from the N-terminus to the C-terminus. The dumbbell shape, consisting of the two linked RecA-like domains that are common to the DBPs, is obtained for both models. (c) Apo-LieIF_trunc/MD_ model with a representation of the conserved motifs and the identified pockets. Pocket P1 is the orange grid. Pocket P2 is the blue grid. Conserved motifs of the DEAD-box family are shown in different colors: Q-motif in red, motif I in yellow, motif Ia in green, GG doublet in yellow, motif Ib in blue, motif II in magenta, motif III in orange, motif IV in red, QxxR motif in blue, motif V in green and motif VI in yellow. The phosphorylation site (T135) observed in an amastigote version of LieIF is shown in magenta.

LieIF is an RNA-dependent ATPase and an ATP-dependent RNA helicase. [9] It shares 53% identity with the mammalian eIF4AI (DDX2A) and 57% identity with eIF4AIII (DDX48), both of which belong to the RNA helicase family of the DEAD-box proteins (DBPs). [31, 32] The amino acids (AAs) sequence of LieIF contains the eleven characteristic motifs of the DBPs. The fact that LieIF is an RNA-dependent ATPase and an ATP-dependent RNA helicase confirms that it belongs to the DBPs family. [9] DBPs are RNA helicases associated with all processes involving RNA, from transcription, translation, splicing, RNA modification to RNA degradation. [33] They share a ∼400 residue-long core region containing the characteristic motifs of the DBPs (Fig 1), and non-conserved flanking regions. Moreover, they all present a dumbbell 3D shape, consisting of two RecA-like domains connected by a flexible linker having variable size and sequence (Fig 2). [34] The N-terminal domain of the conserved core, also called domain 1, contains motifs Q, I, Ia, GG, Ib, II and III. The C-terminal domain, called domain 2, contains motifs IV, QxxR, V and VI. These motifs are important for the biological activities of the DBPs. It has been demonstrated that: (i) motifs I and II from domain 1 are implicated in ATP binding and hydrolysis; (ii) motif III couples ATP and RNA binding and therefore indirectly affects the unwinding activity; and (iii) motifs Ia, GG and Ib in domain 1, and motifs IV, QxxR and V in domain 2 are implicated in RNA binding. [33–41] Domain 2 also is involved in ATP binding through motifs V and VI. Noticeably, ATP binding and hydrolysis, as well as RNA binding and unwinding, are highly dependent on a complex and not yet fully elucidated network of interactions between both domains.

Inter-domain interactions occur mainly when a DBP binds the substrate, which results in a compact closed conformation. In this structure, most of the conserved motifs are located in the cleft formed between the two domains, and they largely interact with Mg^2+^, ATP and RNA ligands. [37–41] RNA bound to the DBP contacts both domains and stabilizes the closed conformation. Inversely, in the absence of ATP or RNA substrates, no inter-domain interactions are observed and an open conformation is adopted. In this case, the two domains present different relative orientations [37, 42–44] due to the linker flexibility. The conformational change (open to closed) occurs when both ATP and RNA are bound. [45, 46] Once the RNA is unwound, ADP and inorganic phosphate are released and the open conformation is adopted. A new catalytic cycle can then take place.

Comparisons of LieIF with yeast eIF4A, a functional homolog of mammalian eIF4A, showed similar enzymatic activities, as one might expect for enzymes involved in the same process. [9] Still, significant differences in the biochemical properties, such as their affinity for ATP and RNA, were observed. [9] Notably, LieIF appears to have a higher affinity for RNA than the yeast protein. LieIF has less affinity (higher K_m_) for ATP and a higher k_cat_ value for the ATPase activity. It has similar affinity for both ADP and ATP, while in yeast and human eIF4A the affinities for ADP are about three times higher. It also has a broader optimum range of divalent cation (Mg^2+^) concentrations for its ATPase reaction. Thus, there were clear differences in the properties between LieIF and yeast eIF4A (and presumably the human protein) that in principle can be used to selectively target the LieIF protein. [9]

At the primary sequence level, the two proteins show the highest divergence in their N-terminal parts. Deleting the most divergent 25 N-terminal residues that are outside the conserved core abolishes the dominant-negative phenotype that LieIF exerts when expressed in yeast. [9] Nevertheless, neither LieIF nor mouse eIF4AI complemented for the loss of the eIF4A-encoding genes in yeast. [9, 47] This suggests the existence of organism-specific interactions between protein partners in the cell that are mediated through the N-terminal sequences. [9] Moreover, it has been proposed that Vasa, a related DEAD-box protein from Drosophila, regulates ATP binding using residues located in the non-conserved amino-terminal sequence. [39] Thus, the importance of LieIF in translation initiation and its significantly distinctive features constituted strong arguments to consider this protein as a potential drug target. Moreover, highly conserved proteins implicated in vital processes are recognized as potential targets for drug discovery. [48, 49]

In this work, we present a computational approach for the *in silico* selection of novel small molecules targeting LieIF followed by a biochemical screening for inhibiting its ATPase activity, and we present evidence for the biological effects of LieIF inhibitors on both *L. infantum* promastigotes and intracellular amastigotes. We used available structure information on the DBPs from the Protein Data Bank (PDB) [50, 51] to build 3D models of LieIF through a comparative modeling approach. We generated open and closed conformation models. We validated their stereochemical quality and their stability in molecular dynamic (MD) simulations. MD trajectories were then used to identify relevant cavities, and two potential binding pockets were selected on the open conformation of LieIF. Virtual screenings (VS) were performed with these pockets and a filtering protocol was set for each pocket using Self-Organizing Maps (SOMs) as a clustering technique. Other chemical, energy-based and geometrical filters were used to select a final set of molecules. We then assessed the effects of these molecules on the ATPase activity of LieIF and its mammalian homolog DDX2A (eIF4AI_Mus_). The most promising hit, **208**, was used as a bait to search and select for 10 chemical analogues that were tested for a potential inhibiting effect on the LieIF ATPase activity and on promastigote viability. The inhibitors affected the promastigotes viability, did not present toxic effects on mammalian cells and reduced the number of amastigotes in the infected cells. This study is a first step towards the validation of LieIF as a potential drug target and identifies chemically related compounds as promising prototypes of novel leishmanicidal compounds.

## Materials and methods

### Molecular modeling

#### Comparative modeling

Protein sequence of LieIF (*LinJ.01.0790/LinJ.01.0800*) was downloaded from the UniProt database (http://www.uniprot.org) under the accession number A4HRK0. A comparative approach was set for the modeling of its 3D structure in five steps using the python library Biskit [52, 53] as follows: (i) The non-redundant (NR) Blast protein database was parsed using PSI-Blast [54, 55] to search for homologous sequences to LieIF. (ii) Templates were searched within deposited protein structures in the Protein Data Bank (PDB) [50, 51] using Blast. Found entries were clustered using BlastClust. From each cluster, the PDB entry with the highest resolution was selected as a template. (iii) The identified templates were sorted into two groups: apo-structures and holo-structures. (iv) For each group, we used T-coffee [56] to perform multiple sequence alignments of LieIF with the template sequences; and to perform multiple structure alignments of the template structures. (v) Based on these alignments (apo, holo), 3D models of LieIF were generated using Modeller 9.8 [57] with its default parameters. Ten models were generated for each group (apo, holo).

For each group (apo and holo), Ramachandran plots of the 10 models were generated using the web server RAMPAGE. [58, 59] The model with the best Ramachandran plot of its group was selected. The selected models were denoted apo-LieIF and holo-LieIF.

A model of the phosphorylated form of LieIF in its unbound form (phos-LieIF) was generated through steps (iv) and (v) of the procedure described above. We used apo-LieIF as a unique template to build the phos-LieIF model.

#### Molecular dynamics simulations

The atomic coordinates for apo-LieIF (unbound) and holo-LieIF (bound) and the mammalian eIF4AI (chain A of the PDB entry 3EIQ) were used as starting structures for MD simulations. The AMBER (Assisted Model Building with Energy Restraints) suite of programs [60] was used for both simulations. The systems were prepared using the Leap module of AMBER. The protein charge was neutralized by adding counterions, and it was solvated by adding transferable intermolecular potentials 3 (TIP3) water molecules. The conformation of the solvated protein was first relaxed through energy minimization. Following minimization, the system was gradually heated from zero to 300 K, with positional restraints on the protein atoms over a period of 0.1 ns. During another 0.1 ns simulation time at 300 K, the positional restraining force constant was gradually reduced from 50 kcal/mol^-1^ Å^-2^ to zero. The simulation system was further equilibrated without any restraints for 0.4 ns followed by a 2 ns recording period (1 fs time step). MD simulations were performed at constant pressure of 1 bar with relaxation time of 2 ps. Solute coordinates were stored every 0.1 ps.

Root mean square deviations (RMSD) of C-*α* atomic coordinates were calculated taking the starting structure of a trajectory as a reference.

#### Pocket search

Cavities were detected on 100 snapshots taken over a 2 ns MD trajectory using an in-house software based on Lee and Richards solvent accessible surface detection algorithm, [61] called *mkgrid*. [62] For each snapshot structure, the space was discretized on a 0.5 Å grid, and the solvent accessible volume was calculated with a 1.4 Å radius probe sphere (also accessing interior cavities). Bulk solvent was defined with a 10 Å radius probe sphere. Cavities were defined as the volume accessible to the solvent, but not to bulk solvent. Remaining void grid points were clustered by connectivity and labeled according to their cluster number to identify individual cavities. Clusters having less than 96 points (12 Å^3^, approximately the volume of a water molecule) were discarded.

We disposed of 100 cavity grids (one per snapshot) and clustered them. We examined the most populated clusters and we manually selected the cavities of interest in the present work based on our knowledge on the DBPs.

### Self-organizing maps

We used an in-house implementation of the Self-Organizing Maps (SOM) algorithm first introduced by Kohonen [63] to analyze the ligand docking poses upon the different virtual screenings that we present below. We trained a 2D periodic map, (Ω_*ij*_)_0≤*i*≤*I*,0≤*j*≤*J*_, with *n* input vectors containing the Euclidean distances between the C_*α*_ of each amino acid defining the targeted pocket and the center of mass of each of the *n* docking poses. The map dimensions *I* and *J* were set to 50. The map was initialized randomly with a uniform distribution preserving the range of values composing the input vectors. The training process was composed of cycles. In each cycle, each input vector was presented once in random order and the map was updated after each presentation. Two phases, similar to that presented by Bouvier [64], were pursued. In the first phase *ϕ* = 1, two training cycles were performed with constant radius and learning rate equal to 36 and 0.5, respectively. In the second phase, three cycles were performed. The radius and the learning rate decrease exponentially from 36 to 1 and from 0.5 to 0, respectively. The decay constant of the exponential, *λ*_*ϕ*_, was equal to the total number of iteration per phase divided by 10.

An efficient way to visualize the SOM map was the unified distance matrix, called the U-matrix. [65] It contained the mean euclidean distance of the map neurons to their respective 26 neighbors. Clusters containing neurons with U-values lower than a given cutoff (in Å) could be defined. They are referred to as clusters with high homogeneity in the present manuscript.

### Virtual screenings

Virtual screenings (VS) of the French Academic Compound Library, called “Chimiothèque Nationale” (CN) [66, 67] were performed on the identified pockets. The version of the database used in this work contained 43407 chemical compounds. All possible stereoisomers were generated for each compound using Corina. [68, 69] This gave 95493 Mol2 records for docking. The receptor and ligands were prepared for docking with Chimera: [70, 71] Gasteiger charges were added to receptor and ligands, and hydrogen atoms were added to the receptor. The *grid* tool from Dock6.0 [72, 73] (UCSF Dock) was used to generate the energy grid. The spheres defining the docking space within the targeted pockets were generated with *sphgen*. Default parameters were used for the docking with Dock 6.4. Twenty poses were generated, when possible, and recorded with their grid-based scores.

AutoDock vina 1.1.2 [74] (ADvina) also was used in this work. It required input files of the receptor and the ligands in pdbqt format. We used the Open Babel converter [75] to generate them from the Mol2 files. Default parameters were used to generate 20 docking poses, when possible, for each chemical compound.

#### Selection protocol for pocket P1

We screened the CN on P1 with Dock. The subset of successfully docked molecules was analyzed with the SOM algorithm presented above. Docking poses corresponding to neurons of the most homogeneous SOM clusters were selected. A threshold of 1.3 Å was used. We only kept poses from molecules respecting the Lipinski rule of five [76]; *i*.*e*., violation of at most one property among the following ones: (i) logP≤ 5; (ii) less than 5 hydrogen bond (Hbond) donors; (iii) less than 10 Hbond acceptors; (iv) a molecular weight MW ≤ 500Da. Then, a geometrical filter was applied. Retained molecules have to count atoms in two small boxes 1.5 Å thick closing each end of the tunnel-shaped cavity of the binding site. The hierarchical chemical clustering function implemented in ICM MolSoft [77] was used to cluster these molecules based on the 2D-pharmacophore method into chemical clusters. Molecules within each chemical cluster were ranked by ascending docking scores. Selection cycles were performed through selecting the best-scored compound from each cluster. Cycles were repeated until all compounds were ranked.

#### Selection protocol for pocket P2

Three docking calculations were performed. First, we screened the CN with Dock on pocket P2. Poses of the successfully docked molecules were clustered with SOMs. Homogeneous clusters defined with a cutoff of 1.3 Å formed a list called SET1. Then, molecules from SET1 were docked on the phosphorylated form of P2 (phos-LieIF) with Dock. The list of molecules successfully docked on this form was called SET2. The last docking calculation was performed with ADvina on the non-phosphorylated pocket P2. Molecules successfully docked constituted SET3. To build the set of selected molecules docked on P2, we considered the intersection between SET2 and SET3. Then, for the diversity sake, we choose the best scored molecules exclusive to SET1 and those to SET3. In view of the length of the tunnel-shaped cavity of P2, the geometric filtering was performed by discarding compounds with less than 50% of their heavy atoms inside a box covering this cavity.

#### Docking of the active molecules

Active molecules were redocked on their respective pocket (P2) using AutoDock 4.2. [78] PDBQT files of the receptor and the ligands were generated by adding all hydrogen atoms, merging non-polar hydrogens and adding gasteiger charges using ADT AutoDock Tools. [78] The 3D grid box was then drawn to include all the residues defining the pocket P2 with dimensions 50 × 45 × 42 (Å) with 0.357 Å spacing. The Grid was then generated using AutoGrid 4. [78] The Genetic algorithm was used for the search step with a ranked cluster analysis of the output. Binding poses with the lowest binding energies were retained and used to calculate atomic pairwise euclidean distance between each ligand and the protein. Distances lower or equal to 3 Å were considered and examined as potential protein-ligand interactions.

### Analogues identification

The most promising hit, identified through virtual and biochemical screenings, and all compounds constituting the Zinc database [79] were decomposed using the circular Morgan Fingerprints [80, 81] as implemented in RDkit. [82] Fingerprints were calculated by decomposition of the compounds into substructures with a user-defined radius limit (set to 2 atoms). A unique integer identifier was assigned to each substructure. Fingerprints consisted in a vector of the substructure identifiers and their number of occurrences in the corresponding compound. To calculate similarity between these fingerprints, the Jaccard similarity criterion was extended to integer values. For that, each integer variable of value, *n*, is replaced by a series of booleans of which the first *n* are set to *true*, the rest being *false*. Hence, for descriptor vectors *A* and *B*, restricted for computation purpose to the *N*_*id*_ integers for which *A*_*i*_ or *B*_*i*_ is/are non-zero, the similarity can be calculated as follows:

$$\begin{array}{c} J\left(A,B\right)=\frac{\sum_{i=1}^{N_{id}}min\left(A_{i},B_{i}\right)}{\sum_{i=1}^{N_{id}}max\left(A_{i},B_{i}\right)} \end{array}$$ (1)

Extended Jaccard similarity was calculated between the bait (compound **208**) and each compound of the Zinc database to rank them from most to least structurally homologous. Among stereoisomers, the compound presenting the closest stereochemistry was selected.

### Compounds

Molecules selected *in silico* were purchased from the corresponding chemists through the French Academic Compound Library System [66], for the minimal costs of their shipping. Minimal quantities necessary for preliminary ATPase assays could be obtained. Compounds 6-*α*/*β*-aminocholestanol (**208**) and 6-*α*-aminocholestanol (**20**) were provided by Université de Caen de Basse-Normandie, Centre d’Études et de Recherche sur le Médicament de Normandie (CERMN), UFR des Sciences Pharmaceutiques, under the references MR26628 and MR26620, respectively. Compound 6-ketocholestanol (**48**) was purchased from Sigma-Aldrich (St. Louis, MO, USA) under the reference K1250. The other 8 compounds also were purchased from Sigma-Aldrich. Stock solutions of all compounds were prepared at 10 mM in dimethyl sulfoxide (DMSO; Sigma-Aldrich).

### Enzymatic validation

#### Expression and purification of recombinant LieIF and eIF4AI_Mus_

The *Rosetta Escherichia coli* strain (Novagen) was used for the expression of His-tagged recombinant LieIF and mouse eIF4AI (eIF4AI_Mus_) proteins using already described constructs [9]. Protein expression was performed as previously described, except that cultures were induced with 0.5 mM IPTG. [9, 83] Cells were collected by centrifugation and stored at -20°C until needed. Pellets were resuspended in 10 ml of buffer A (Tris-HCl, pH 7.5, 300 mM NaCl) supplemented with 10 mM imidazole and 0.02% NP40 (lysis buffer). Lysozyme (10 *μ*g/ml, Sigma-Aldrich, St. Louis, MO, USA) and 1x of complete protease inhibitor EDTA-free cocktail (Roche, Indianapolis, USA) were added. The mixtures were kept on ice for 30 min. Then, cells were ruptured by sonication four times for 20 seconds with a Cell Disruptor W375 (Heat System-Ultrasonic, Plainview, N.Y., USA) using a microprobe at a setting of 5 and a duty cycle of 60%. The material was centrifuged for 30 min at 15,000 rpm in a JA-20 rotor (Beckman Coulter, Villepinte, France) at 4°C, and the supernatant was loaded onto a 1 ml nickel-nitrilotriacetic acid (Ni-NTA) agarose column (Ni-NTA, Qiagen, Hilden, Germany) equilibrated with the lysis buffer. The column was washed with 20 ml buffer A supplemented with 25 mM imidazole and 0.02% NP40 (wash buffer 1), and then with 20 ml buffer A supplemented with 25 mM imidazole (wash buffer 2). Proteins were eluted with buffer A supplemented with 100 mM imidazole (elution buffer). The purity of the proteins was checked on 12% SDS-PAGE gels that were stained with Coomassie blue. Purified proteins were stored at -80°C after making 50% in glycerol (w/w). The proteins concentrations were determined using the Bio-Rad Protein Dye Assay (Biorad, Munich, Germany).

#### ATPase assays and analysis

We used a colorimetric assay based on Malachite green as previously described. [9] We optimized the previously published conditions for the LieIF and eIF4AI_Mus_ ATPase assays to fit microtiter plates and the screening conditions [9] by varying the quantities of proteins, ATP and magnesium acetate, and by testing different sources and concentrations of RNA. Each reaction was done in 50 *μ*l. The final reaction buffers contained 50 mM potassium acetate, 20 mM MES, pH 6, either 5 mM or 1 mM magnesium acetate for LieIF or eIF4AI_Mus_ respectively, 2 mM dithiothreitol (DTT), 0.1 mg/ml bovine serum albumin (BSA) and 0.34 mg/ml whole yeast RNA (Type XI, Sigma). Reactions were incubated at 37°C for various times up to 45 min, and were stopped by adding 5 *μ*l of 0.5 M ethylenediaminetetraacetic acid (EDTA) at pH 8. In experiments testing the compounds, samples dissolved in DMSO were added to the reaction in different concentrations. The final concentration of DMSO in the reaction was 10% (v/v). The reaction rates were determined by a linear regression fit of the initial, linear phase of the curves. Reaction rates were determined in three independent experiments for each compound concentration. Data were analyzed with Kaleidagraph (Synergy).

#### Statistical Z’-analysis

The Z’-factor is a statistical parameter used to measure the quality of a screening assay. [84] For the biochemical screening, reactions were done with and without proteins as positive and negative controls, respectively. All assay conditions were identical to those described above. Positive controls were designed to replicate a positive hit in an inhibitor screen. Negative controls were designed as no enzymatic reactions. The phosphate released was measured after 45 min. Statistical analysis was performed using the equation:

$$\begin{array}{c} Z^{\prime }=1-\frac{3*(\sigma^{+}+\sigma^{-})}{\left(\mu^{+}-\mu^{-}\right)} \end{array}$$ (2)

where *μ*^−^ and *σ*^−^ correspond to the mean absorbance and standard deviations respectively, for each reaction without enzyme (no activity), and *μ*^+^ and *σ*^+^ are the mean absorbance and standard deviations, respectively, for reaction wells in the presence of enzyme. The Z’-factor values above 0.5 were considered significant and corresponding plates were retained. [84]

#### Biochemical screens

Biochemical screening experiments were performed in 96-well plates. Column 1 and 12 contained full signal controls (no inhibitor) and background controls (no enzyme): wells A1 to D1 and E12 to H12 corresponded to negative controls; E1 to H1 and A12 to D12 corresponded to positive controls. Compounds were added in columns 2 to 11, at a 500 *μ*M final concentration. ATPase test reactions were done in three independent experiments. The phosphate released was measured after 45 min. Results were reported as weighted means. Z’-scores were calculated for each plate from the full signal and background controls. [84] Plates with Z’-values below 0.5 were discarded. The inhibition of ATPase activity was calculated as follow: [85]

$$\begin{array}{c} Percentage\;of\;inhibition=\left(\mu^{+}-OD_{630nm}\;for\;compound\;X\right)/\left(\mu^{+}-\mu^{-}\right)*100 \end{array}$$ (3)

Selected hits were further tested with kinetic assays. Results were reported as ATPase reaction velocities. Therefore, the relative ATPase reaction rate in the presence of increasing concentrations of the compounds were plotted as a function of compound concentrations and the 50% inhibitory concentration (IC_50_) values were determined.

### Biological validation

#### Cell and parasite cultures

The human myelomonocytic cell line, THP-1 was ordered from the American Type Culture Collection (ATCC, TIB-202). [86] Cells were maintained in RPMI 1640/Glutamax-I media (Gibco BRL, Germany) supplemented with 10% heat-inactivated fetal calf serum (Gibco, Germany) plus penicillin G (100 U/ml) and streptomycin (100 g/ml). THP-1 cells were differentiated to macrophages after their treatment with 20 ng/ml phorbol 12-myristate 7-acetate (PMA) (Sigma, St. Louis, MO, USA) for 48 h at 37°C, 5% CO_2_. [87] The viability of THP-1 mature macrophage-like cells was determined to be > 97% by the Trypan blue dye exclusion assay. The MON1 *L. infantum* laboratory strain LV50 originating from a visceral leishmaniasis case (Laboratory of molecular epidemiology and experimental pathology, Institut Pasteur de Tunis) was used to infect the PMA activated THP1 cells as well established in the laboratory. To enhance the infection rate, promastigote cultures were preconditioned as previously described. [87] Briefly, 10^6^ promastigotes/ml were inoculated in RPMI-1640 media supplemented with 2 mM L-glutamine, 100 U/ml penicillin, 100 U/ml streptomycin, and 10% (v/v) heat-inactivated fetal bovine serum at 22°C. Promastigotes were collected at the beginning of the stationary phase (day 5), counted, centrifuged, seeded at 10^6^/ml in 10 ml of acidic complete media (pH = 5.4) and incubated overnight at 22°C. The next day the parasites were collected and used in the infection assays.

#### MTT assays to test parasite viability and toxicity on macrophages

The effect of the selected chemical compounds on the viability of *L. infantum* promastigotes was evaluated by a colorimetric MTT (3-(4,5-dimethylthiazol-2yl)-2,5-diphenyl tetrazolium bromide) assay that consisted in a reduction of tetrazolium salt to a soluble crystal (blue formazan) by the succinate dehydrogenase activity of mitochondria in living cells, which can be quantified by spectrophotometry as previously described. [88] Briefly, 90 *μ*l of promastigotes harvested from the stationary growth phase were added to a 96-well culture plate (5 × 10^5^ cells/well) with 10 *μ*l of various concentrations of the selected compounds (to obtain a final concentration of 1.5-100 *μ*M) and incubated at 25°C for 24h. After incubation, 20 *μ*l of MTT solution (5 mg/ml) were added to each well and incubated at 25°C for 4h. Then, 150 *μ*l of DMSO was added to each well to dissolve the blue formazan and the optical density (OD) was measured at 560 nm with a microplate reader (MULTISCAN, Labsystems). Mock treated-promastigotes in complete medium (with no drug, 1% DMSO) were used as positive control. Stock solutions of the compounds prepared for these experiments contained 100% DMSO. All subsequent dilutions were freshly made with RPMI 1640 (final concentration of 10% DMSO). Thus, all compounds, as well as the positive control, were tested at a final concentration of 1% DMSO per well.

For the cytotoxicity assay, we used the THP-1-derived macrophages (50,000 cells/well) seeded in 96-well plates and treated with serial concentrations of compounds (final concentrations of 1.5-100 *μ*M) for 24h. After that, an MTT assay was performed as previously described to determine cell viability. [88] Thus, 20 *μ*l of MTT solution (5 mg/ml) were added to each well and incubated at 25°C for 4h. Then, 150 *μ*l of DMSO were added to each well to dissolve the blue formazan and the optical density (OD) was measured at 560 nm with a microplate reader (MULTISCAN, Labsystems).

#### Analysis of MTT assays

The promastigotes viability was expressed as the percentage of the viable promastigotes in treated conditions relative to the 1% DMSO mock-treated promastigotes. The IC_50_ values were calculated for all the tested compounds by interpolation. The cell viability of THP-1-derived macrophages was expressed as the percentage of the viable cell number in treated cells relative to the THP-1-derived macrophages treated with 1% DMSO. The 50% cytotoxic concentrations (CC_50_) were calculated for all the tested compounds by interpolation. All experiments were repeated three times in triplicate for each compound concentration. Absorbance measured was used to reflect promastigote or cell viability using the following formula:

$$\begin{array}{c} \%\;cell\;viability\;=[\;\left(OD_{test}-OD_{blank}\right)/\left(OD_{control}-OD_{blank}\right)]*\;100 \end{array}$$ (4)

#### Macrophage infection

After careful washing of PMA-treated THP-1 cells (50,000 cells/well) with warm serum-free RPMI-1640 (∼ 37°C) media, 50 μl of the diluted *L. infantum* promastigote culture were added at the optimized MOI (10:1). Control wells of THP-1 cells without the parasites, and THP-1 cells with the parasites, were set up in each 8-well Labtek slides. The slides were incubated at 37°C, 5% CO_2_ overnight. After incubation, infected wells were washed at least 3 times with serum-free RPMI-1640 media to ensure complete removal of non-engulfed promastigotes, and then they were incubated in presence of different concentrations of compounds for 24 h. Control groups were incubated in supplemented RPMI medium containing 1% DMSO. Cells were then fixed and stained with RAL 555 rapid stain kit (May-Grünwald Type) (Cell Path, Newtown, UK) following the manufacturer’s instructions. The number of infected cells per 100 macrophages and the total number of parasites per 100 cells observed were determined by light microscopy under immersion oil (1000X) by counting at least 100 cells per well. Three independent experiments were conducted for each compound concentration. These values allowed us to determine the mean values and standard deviations of the percentage of infected cells and the mean amastigote number per cell. To account for the overall parasite load, an Infection Index was calculated by multiplying these two values; then, to assess the infection inhibition by the compounds, the percentage of Inhibition Parasite Index (IPI) was determined as: *IPI* = 100 − ((*Infection Index in treated cells*/*Infection Index in untreated cells*) * 100).

The amastigote IC_50_ values were calculated for all tested compounds. Selectivity index was also determined for each compound as follows: *SI* = *CC*_50_/*IC*_50_. Data were analyzed with Kaleidagraph (Synergy). The statistical significance of differences was determined with the Student’s t-test (* *p* < 0.05; ** *p* < 0.01).

## Results

### LieIF 3D models

We used comparative modeling to generate 3D models of LieIF in two different states. Ten templates were used for the open form (ligand-free), and seven templates were used for the closed form (substrate-bound; Table 1). Eight out of ten ligand-free templates and four out of seven substrate-bound ones had identity rates (IR) between the protein target and its templates above the twilight region, as needed for robust model construction (*i*.*e*., *IR* ≥ 30%, Table 1). [89–91] Templates having IR< 30% were nevertheless kept for diversity sake. LieIF models were built and assessed for their robustness.

**Table 1:** Templates used to build the 3D models of LieIF (PDB ID and chain ID) listed with descending identity rates.

N°	PDB_ID	Fragment	Function	Organism	Resolution(Å)	Identity(%)
1	1FUK_A	230–394	Yeast eIF4A	*Saccharomyces cerevisiae*	1.75	57.69
2	3FHO_B	1–503	RNA helicase DBP5	*Schizosaccharomyces pombe*	2.80	40.07
3	2KBE_A	71–296	RNA helicase DBP5	*Saccharomyces cerevisiae*	NA(*)	39.55
4	1HV8_B	1–367	MjDEAD RNA helicase	*Methanocaldococcus jannaschii*	3.00	36.29
5	1XTI_A	46–428	RNA helicase P47	*Homo sapiens*	1.95	34.68
6	2JGN_C	408–579	RNA helicase DDX3X	*Homo sapiens*	1.91	34.39
7	1Q0U_B	2–219	BSTDEAD N-terminus	*Geobacillus stearothermophilus*	1.85	34.34
8	2Z0M_A	1–337	Hyp. RNA helicase	*Sulfolobus tokodaii*	1.90	31.50
9	3EAQ_B	215–426	RNA-dependant ATPase	*Thermus thermophilus*	2.30	28.93
10	3I32_A	218–517	RNA-dependant ATPase	*Thermus thermophilus*	2.80	27.27
11	2XB2_X	1–411	eIF4A-III (DDX48)	*Homo sapiens*	3.40	56.99
12	2HYI_I	1–411	Prob. RNA helicase DDX48	*Homo sapiens*	2.30	56.70
13	2GXQ_A	1–207	RNA-dependent ATPase	*Thermus thermophilus*	1.20	37.93
14	3FMO_B	1–300	RNA helicase DDX19B	*Homo sapiens*	2.51	37.33
15	2DB3_D	200–623	RNA helicase VASA	*Drosophilia melanogaster*	2.20	27.74
16	3DKP_A	139–381	Prob. RNA helicase DDX52	*Homo sapiens*	2.10	27.35
17	3I5X_A	37–597	RNA helicase MSS116	*Saccharomyces cerevisiae*	1.90	26.56

From 1-10: templates used to build apo-LieIF. Mean identity rate value is 36.5 ± 8.1%.

(*) NMR experiment.

From 11-17: templates used to build holo-LieIF. Mean identity rate value is 38.7 ± 12.3%.

Ten models were generated for each state and their Ramachandran plots were assessed (see S1 Table for a summary) in order to help us select the most reliable model for each state. For the ligand-free models, we chose model N°4 presenting 95.3% residues within the favored region (98% expected); 3.0% residues within the allowed region (2.0% expected) and 1.7% residues within the outlier region (0.0% expected). For the substrate-bound, model N°3 was considered as the best structure with 97.5% of the residues within the favored region, 2.0% within the allowed region and only 0.5% within the outlier. These two structures were selected and will be referred to as apo-LieIF and holo-LieIF, respectively (Fig 2). They presented different conformations as expected; apo-LieIF resembled the open conformation of the DBPs and holo-LieIF was quite compact representing the closed conformation (see their Ramachandran plots in S1(a) and S1(b) Fig, respectively). Both apo-LieIF and holo-LieIF presented unstructured termini. These regions were described in many DBPs as intrinsically disordered. [36] On some of the unbound models of LieIF, the N-terminal sequence tended to fold into the inter-domain cleft. Hence, we considered their structures as unreliable, since they were obtained through a comparative modeling but lacked reliable alignments with the templates’ termini. In fact, many templates had been truncated (Table 1) for experimental reasons, and high divergence was observed between LieIF models and their templates at the N- and/or C-terminal regions. Thus, the termini structures were removed as a conservative measure prior to MD simulations and VS calculations. Twenty-four N-terminal and seven C-terminal residues were removed. Among them, 4 out of a total of 12 and 1 out of 8 were in the allowed region of the Ramachandran plots of apo-LieIF and holo-LieIF, respectively. For the outliers, these figures were 3 out of 7 and 0 out of 2, respectively. Truncated structures of LieIF [AA 25-396] were denoted apo-LieIF_trunc_ and holo-LieIF_trunc_. The resulting percentages of favored, allowed, outliers amino acids were 97.4, 1.5, 1.1%, and 99.6, 0.4, 0.0% for apo-LieIF_trunc_ and holo-LieIF_trunc_, respectively.

### Identifying potential docking pockets

To verify the stability of the structures and to probe their local relaxations, we ran MD simulations for apo-LieIF_trunc_ and holo-LieIF_trunc_ during 2 ns. As we did not mean to study longer-term protein motion, relatively short simulation times were chosen. For comparison purposes, we performed similar calculations for the mammalian eIF4AI using chain A of the PDB entry 3EIQ (3EIQ_A). Holo-LieIF_trunc_ RMSD varied within 0.5 and 2.0 Å, a fairly stationary evolution through time, thus indicative of its stability. In contrast, apo-LieIF_trunc_ displayed RMSD within 0.5 and 4.5 Å, presenting increasing values through the trajectory (S2 Fig). These somewhat larger variations could seem wide, but in fact they depict higher flexibility of the protein in its unbound form due to the presence of a flexible linker between the two fairly independent domains (Fig 2; apo-LieIF_trunc_). In this latter case, the trajectory would embed higher conformational diversity. Noticeably, similar variation could be observed with the crystal structure of the mammalian protein (3EIQ_A), leading us to consider apo-LieIF_trunc_ as a more relevant state of the protein as compared to holo-LieIF_trunc_ (S2 Fig).

Substrates of eIF4AI bind within the inter-domain cleft, which makes the active site definition too fuzzy and large for accurate docking simulations. Thus, we used the MD trajectories of both states of LieIF and 3EIQ_A to search for pockets that may have functional relevance for the parasite protein, but no equivalent on the mammalian counterpart. The compact structure of holo-LieIF_trunc_ presented small fluctuations, and no interesting cavities could be detected (S3 Fig). Conversely, apo-LieIF_trunc_ presented multiple cavities including the inter-domain cleft. A clustering step enabled us to identify the cavities consistently present during the trajectory (2ns). Two pockets were manually selected based on our knowledge on DBPs. They were both present on snapshot N°19 of the MD trajectory of apo-LieIF_trunc_, and they have no equivalent on the mammalian protein (S3 Fig). This particular structure was considered for further analysis, and it will be denoted as apo-LieIF_trunc/MD_.

The first pocket was located at the beginning of the truncated protein (Fig 2). It was constituted by 17 residues [AA 27-33, 38, 41-42, 46-50, 53-54]. It had a volume of 132 Å^3^, and it will be referred to as P1. Although it had a small size, it was selected as a potential druggable pocket of LieIF for two reasons. First, P1 was spatially close to the divergent 25 N-terminal residues of LieIF that were responsible for the dominant negative phenotype of LieIF in yeast that leads to growth impairment. [9] Hence, it has a significant potential to create a binding site for *Leishmania*-specific compounds. The second reason was the fact that it contained the Q-motif [AA 45-53], which is an adenine recognition element with features common to all ATP-dependent helicases, and it may play the additional role of regulating ATP binding. [33, 36] Moreover, the Q-motif was at the interface between the two domains constituting LieIF. Inter-domain interactions are known to be important in ATP binding and hydrolysis in many DBPs. [39, 92] A small molecule that binds to P1 may interfere with inter-domain interactions by steric hindrance or by impeding conformational changes necessary for ATP binding and/or hydrolysis. These elements suggested that P1 could be a suitable specific inhibitory binding site in association with the non-conserved amino terminus of LieIF.

The second pocket, referred to as P2, also was located on domain 1 (Fig 2) and was constituted by 33 residues [AA 104, 107-108, 111-112, 115, 125-137, 139-140, 142-144, 154, 156-157, 160-161, 164-167]. Thus, P2 contains residues from: (i) motif Ia [AA 102-107], (ii) motif Ib [AA 154-158], (iii) the variable loop containing the GG doublet [AA 133-134] and the THR135 residue, which is the only phosphorylation site known for *L. infantum* LieIF [93], and (iv) a sequence insertion that is specific to LieIF [AA 125-127]. The conserved motifs Ia, Ib and the doublet GG are implicated in RNA binding in the DBPs. [15, 37, 39, 40, 94] The sequence insertion that is unique to LieIF may increase the probability of identifying selective molecules, as has been the case for other *Leishmania* proteins. [95] In fact, residues [S125, K126, F127] of this insertion constitute a part of a long loop connecting the *α*-helices containing motifs Ia and Ib. Residues corresponding to this loop in other DBPs have different secondary structures depending on the protein. For example, DDX2A (3EIQ), yeast eIF4A (1FUU), DDX48 (2XB2) and Vasa (2DB3) present two *β*-sheets separated by an *α*-helix, while DDX19 (3G0H, 3FMO) and yeast DBP5 (2KBE) present only two *β*-sheets separated by a long variable loop at this particular region. Thus relevant differences were observed on the structural and sequence levels for different DBPs around residues corresponding to P2 on LieIF. In addition, the tunnel-shape of P2 and its size (364 Å^3^) were among the properties that led to its selection as a promising and potentially specific druggable pocket.

### Virtual screenings and molecules selection

In order to select for potential LieIF ATPase inhibitors, we proceeded to a virtual screening of the CN library targeting pockets P1 and P2. Dock was used for docking calculations. Then, a clustering step using Self-Organizing Maps (SOMs) was performed on the VS results. This step permitted us to identify clusters of consensual docking poses. To reduce the number of compounds within those clusters, we used other filters according to the pocket and based on drug likeness properties, low (favorable) energy of interaction with the protein, pose geometry or chemical diversity.

For pocket P1, only 19013 compounds were successfully docked out of the initial set (95494 compounds). The SOM analysis revealed a map with two low U-valued clusters presenting low (favorable) docking scores (S4(a) and S4(b) Fig). These clusters contained 2921 compounds that were mainly small with low molecular weights. This is essentially due to the relatively small size of the pocket (132 Å^3^). Since oral drugs against VL are highly recommended, we filtered them according to the Lipinski “Rule of Five” and to a geometrical sieve which reduced the set down to 783. We clustered them according to their chemical structures and ranked them according to their docking scores within each chemical cluster. From each cluster, the two best-scored molecules were selected, when more than one compound occurred. So, we retained a selection of 131 consensual, chemically-diverse drug-like molecules, correctly docked inside the pocket and well scored (according to Dock grid-based scores).

For pocket P2, we also docked the CN molecules using Dock. The SOM analysis revealed three homogeneous clusters with low docking scores (S4(c) and S4(d) Fig) that contained 12408 compounds (*SET*1). This represented a larger number of docked molecules as compared to P1, and it is due to the large size of this pocket. As P2 contained the THR135, a phosphorylated site of an amastigote version of the LieIF protein, we investigated the effect of such a post-translational modification on the docking of these molecules. We docked the molecules of SET1 on the phosphorylated form of LieIF and only 6712 (∼ 54%) molecules were successfully docked (SET2). A shift to positive docking scores was observed compared to the docking on the non-phosphorylated P2 (S5 Fig). This indicated a global negative impact of the phosphate group on the docking results.

SET2 contained a large number of molecules to be tested *in vitro*. In order to optimize the chance to select relevant molecules interacting with both forms of the protein, we performed a third docking calculation targeting the non-phosphorylated P2. A second docking algorithm was used (ADvina) to screen the CN. It uses a different searching algorithm and a different scoring function compared to Dock. This would permit us to perform a selection with no algorithm-related bias. The SOM analysis revealed a map with three homogeneous clusters (S4(e) and S4(f) Fig) containing 12298 compounds (SET3). The intersection between SET2 and SET3 contained 155 molecules. Through the geometric filter, we eliminated 11 molecules. Thus, the remaining 144 compounds constituted a set of molecules with consensual docking poses according to two different searching algorithms and presenting good docking scores according to two different scoring functions.

For the sake of diversity, two additional sets of molecules not included in the intersection (SET2 ∩ SET3) were constituted. Fifteen molecules exclusively docked with Dock, with low, favorable docking scores and passing the geometrical filter were selected (SET1 but not SET2 or SET3). Similarly, fifteen molecules docked exclusively with ADvina with low docking scores and good poses were selected (SET3 but not SET1 or SET2). Thus, 174 molecules were selected for P2 as potential hits. This final set contained 144 consensually docked molecules through the three VSs and 30 molecules chosen for their best docking scores (Dock or ADvina scores). Finally, 305 molecules (screened against P1 and P2) were selected and purchased at the French Academic Compound Library [66, 67] for experimental validation.

### Selection and characterization of ATPase inhibitors of LieIF

**Fig 3:**
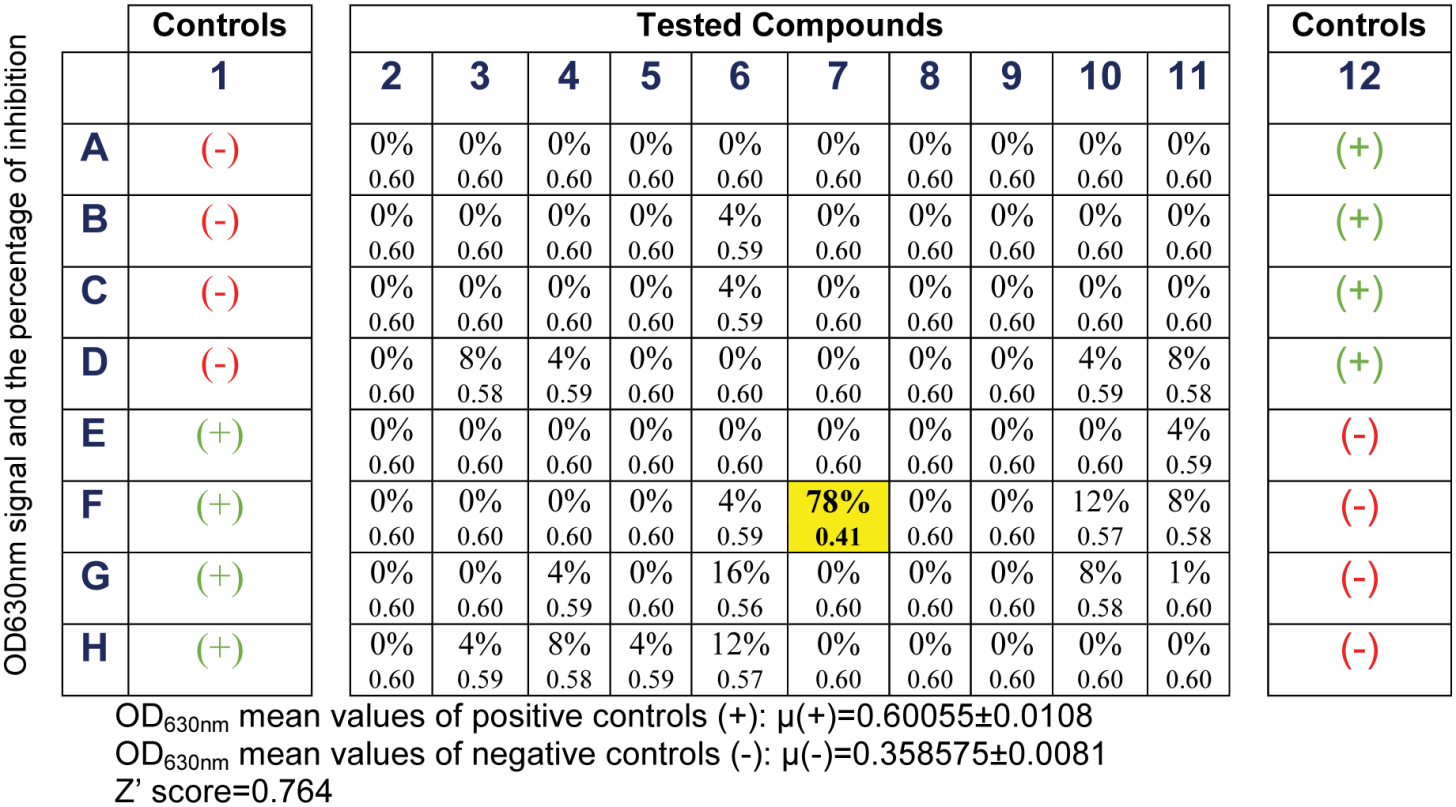
Representative results for one 96-well screening plate. OD_630nm_ values obtained with the malachite green ATPase assay. Columns 1 and 12 correspond to controls with LieIF (“+”; activity) or without LieIF (“-”; no activity). For each tested compound, the figure shows both the OD_630nm_ signal and the percentage of inhibition relative to the control wells. Compounds inhibiting OD630nm signal are highlighted in yellow.

In order to select the compounds that will experimentally inhibit the ATPase activity of LieIF, we established screening assays where both the purified recombinant LieIF and eIF4AI_Mus_ were tested for their ATPase activity in the presence of commercially-available, total yeast RNA with a colorimetric assay based on molybdate Malachite Green that measures the free phosphate released. [9] We performed the screens monitoring the ATPase efficiency in the presence of 500 *μ*M of the compounds in 96 well plates in three independent experiments. We used this concentration in order to enhance the chances of observing inhibition or stimulation of the compounds because we used a relatively high protein concentration in the assays (around 1 *μ*M); high protein concentrations were needed because of the relatively weak RNA-dependent ATPase activities of eIF4A-like proteins. [36] Through the biochemical screen, we calculated the percentage of inhibition of each compound and we detected four signals of inhibition of LieIF corresponding to structurally unrelated molecules; two docked on P1 and two on P2. We show the results for one representative screening plate (Fig 3), where the Z’-score of 0.76 confirmed the quality of the screen. Due to the lack of sufficient amounts of three of the compounds, only compound **208** was further characterized. We also obtained higher amounts of this compound from the corresponding chemists to be able to proceed with further enzymatic and biological experiments.

**Fig 4:**
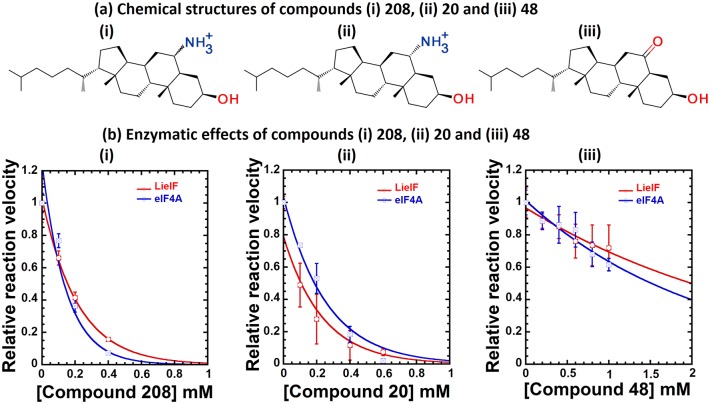
Relative reaction rates of the two proteins in the presence of increasing concentrations of compounds 208, 20 and 48. (a) Chemical structures of the three analogous compounds identified as inhibitors of LieIF. (b) Points with error bars on each curve represent the mean and standard deviations of three independent measurements made using the compound concentrations in the 0 to 1 mM range. The relative reaction velocities were normalized to 1 in the absence of inhibitor to facilitate comparisons. Data were fit to an exponential decay. The relative reaction rate of LieIF (in red) and eIF4AI_Mus_ (in blue) in the presence of increasing concentrations of the compounds were plotted as a function of compound concentrations. The compounds clearly presented different kinetic properties according to the proteins.

Compound **208**, an epimeric mixture (*α*/*β*: 84%/16%) called 6-*α*/*β*- aminocholestanol (Fig 4(a)) [96], was identified within the set of compounds that successfully docked on pocket P2 by both Dock and ADvina. In order to characterize the effect of the compound on the ATPase activity, we performed time courses for the ATPase activity of LieIF and eIF4AI_Mus_ (∼ 1*μ*M) at different concentrations of the compound in the 0 to 1 mM range, in the presence of 1 mM ATP and saturating concentrations of RNA. The amount of ATP hydrolyzed for both proteins increased in a time-dependent manner and the corresponding ATPase reaction rates for each compound concentration were determined and plotted (Fig 4(b-i)). The relative reaction velocity of the ATPase activity decreased in the presence of increasing amounts of the compound in a dose-dependent manner. The IC_50_ values were interpolated for the inhibition of the ATPase activity of 1 *μ*M of LieIF and eIF4AI_Mus_, and we obtained IC_50_ values of 150 ± 15 *μ*M and 115 ± 25 *μ*M, respectively (Fig 4(b-i)). For comparison, the K_m_ reported for ATP in similar reactions were higher than our IC_50_ values (350 ± 120 *μ*M for LieIF and 250 ± 90 *μ*M for yeast eIF4A). [9]

Next, as a proof of concept, we identified chemical analogues of compound **208** to test their effect on LieIF. Nine commercially available analogues of **208** were identified and purchased (Sigma Aldrich, S6 Fig). Moreover, the 6-*α*-aminocholestanol (**20**) could be obtained from the chemists that provided us with the **208** epimeric mixture (S6 Fig). All ten molecules were screened at 500 *μ*M for their effects on the ATPase activity of LieIF and eIF4AI_Mus_. Two molecules (**20** and **48**) demonstrated inhibition of the ATPase activity of LieIF and eIF4AI_Mus_ (Fig 4(b-ii) and 4(b-iii)). Structures of compounds **20** (6-*α*-aminocholestanol) and **48** (6-ketocholestanol) are shown in Fig 4(a).

Therefore, we further characterized these molecules as we did for **208** using time course experiments testing different compound concentrations in the 0-1 mM range: 0, 100, 200, 400 and 600 *μ*M for **20**; and 0, 200, 400, 600, 800 and 1000 *μ*M for **48**. We also determined relative reaction velocities and interpolated IC_50_ values. Compound **20** showed a comparable activity to **208** with IC_50_ values of 160 ± 25 *μ*M and 185 ± 25 *μ*M for LieIF and eIF4AI_Mus_ respectively (Fig 4(b-ii)). On the other hand, compound **48** showed a lower activity. It inhibited the ATPase activity of 1 *μ*M of LieIF and eIF4AI_Mus_ with IC_50_ values up to 1 mM (Fig 4(b-iii)). All compounds presented different kinetic properties according to the proteins (Fig 4).

The least effective compound (6-ketocholestanol) presents a ketone group replacing the amino group on carbon C6 as compared to **208** and **20** (Fig 4(a)). Interestingly, all 8 non-active analogues lacked this group at this possition. Even a nitro group could not ensure activity (S6 Fig). Thus, the amino group appeared important for the inhibitory activity.

### Refinement of the binding modes of LieIF inhibitors

In order to gain insights into the potential binding modes and affinities of the three hits on LieIF, we performed further docking calculations targeting their plausible binding site, pocket P2. Epimers of the mixture **208** were considered separately, as the 6-*α*-aminocholestanol, represented by compound **20**, and the 6-*β*-aminocholestanol. Docking scores and estimated K_i_ values were obtained for each hit. Different binding modes were obtained for the three molecules (S7 Fig).

**Fig 5:**
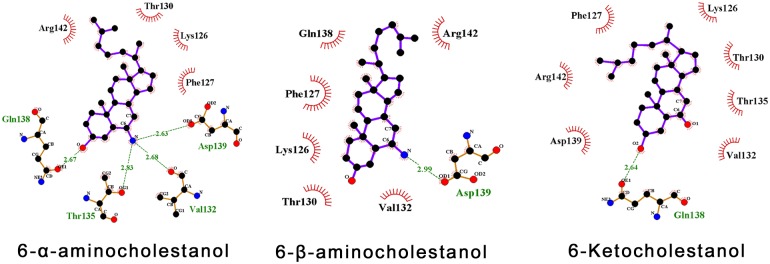
Interaction diagrams of compounds 6-*α*-aminocholestanol (20), 6-*β*-aminocholestanol and 6-ketocholestanol (48) (from left to right) with pocket P2 on apo-LieIF, in the non-phosphorylated form. Hydrogen bonds are shown in green dashed lines along with their length in Å. Residues establishing hydrophobic interactions with the ligands atoms are shown in red incomplete circles. Ligand atoms of the compound that are implicated in these hydrophobic interactions are also surrounded by red sticks.

As a special interest in the amino/ketone group on carbon C6 arose through the ATPase assays, we investigated its potential interactions with the pocket residues. Compound **20** appeared to be the most potent compound according to its estimated K_i_ (221.6 nM) and the free energy of its binding (-9.1 kcal/mol) to LieIF (Table 2). Noticeably, the amino group of compound **20** interacted with the phosphorylation site of LieIF (T135) through H-bonds (Table 2, Fig 5), while the ketone group of compound **48** established hydrophobic interactions with T135 (Fig 5).

**Table 2:** Summary of the docking simulations results of the three hits on LieIF, Phos-LieIF and the mammalian eIF4AI (3EIQ_A).

Compound		6-*α*-aminocholestanol	6-*β*-aminocholestanol	6-ketocholestanol
Estimated free energy of binding	LieIF	-9.1	-7.2	-7.7
	Phos-LieIF	-6.9	-6.6	-7.9
	eIF4AI	-5.5	-3.5	-4.1
Estimated K_i_ (*μ*M)	LieIF	211.6 10^−3^	5.3	2.2
	Phos-LieIF	8.9	14.5	1.7
	eIF4AI	94.5	2.6 10^3^	973.6

All three hits also were docked on phos-LieIF and exhibited either higher or comparable docking scores and K_i_ estimations to those we obtained with the non-phosphorylated form of LieIF. Docking poses on both forms of LieIF presented significantly different interactions between the compounds and the protein residues, suggesting an important impact of the phosphate group on T135 on the pocket properties, which would directly impact the binding of the inhibitors. This confirmed our initial interest in the phosphorylated form of the pocket P2 as a differentiating target in the virtual screening. The best interactions were predicted with the non-phosphorylated form of LieIF, and the highest affinity with compound **20** (6-*α*-aminocholestanol).

Many residues interacting with compound **20** were non-conserved in the mammalian eIF4AI. We performed the same docking calculations targeting a site equivalent to P2 on the mammalian eIF4AI structure, but pockets were different/absent in the site region (S3 Fig). A significant shift to higher docking scores was obtained (Table 2) as compared to LieIF. As no equivalent pocket was detected on the mammalian eIF4AI and taking into consideration the qualitative differences between the kinetics obtained with LieIF and eIF4AI_Mus_ (Fig 4), we hypothesized that the binding modes and sites of the inhibitors differ between both proteins.

### Leishmanicidal activity against promastigotes and amastigotes

**Fig 6:**
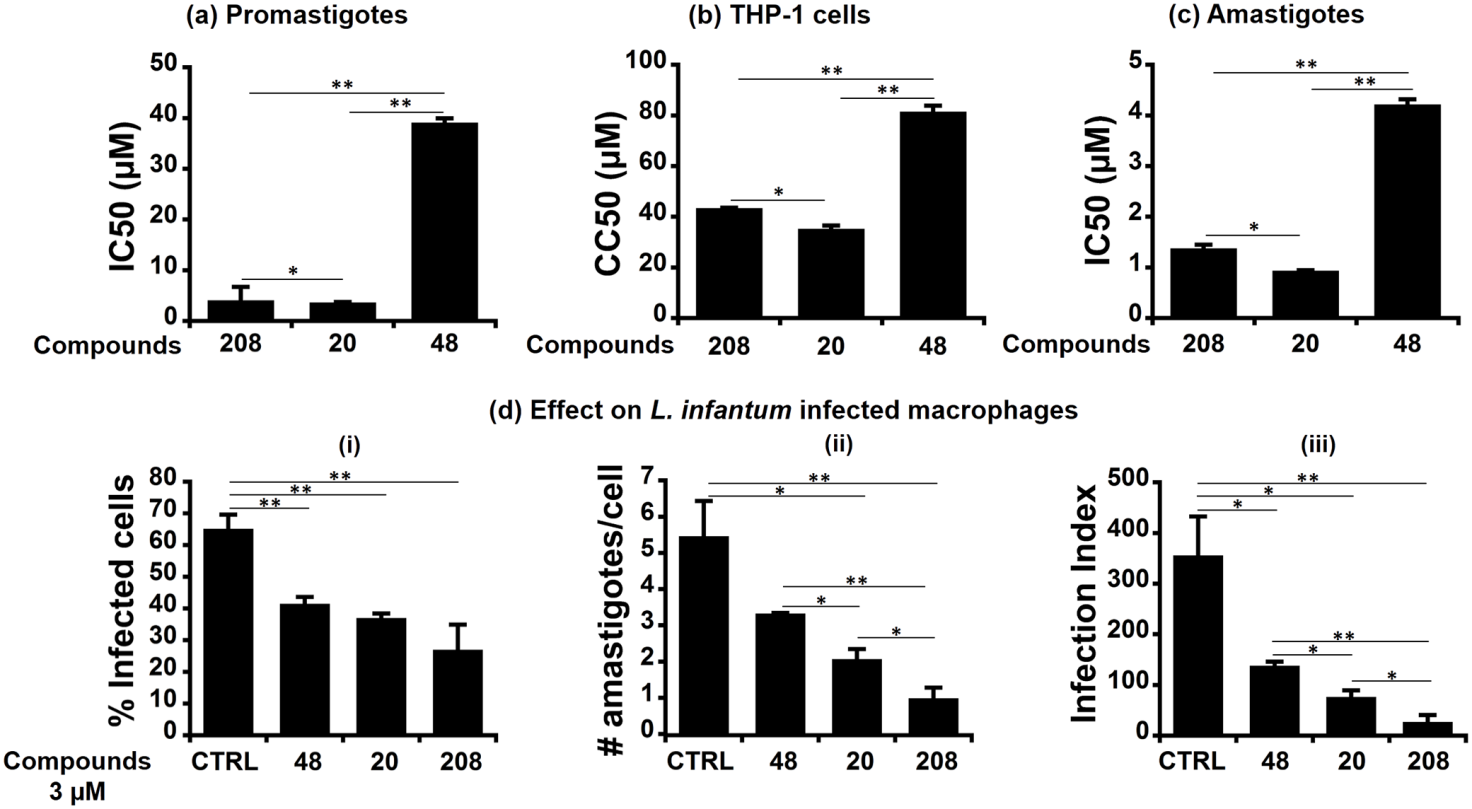
Hits 208, 20 and 48 impaired promastigote viability and amastigote survival in a dose-dependent manner. (a) IC_50_ measured on promastigote viability: Stationary phase parasites were plated in 96-well plates at a final parasite density of 5x10^6^ parasites/ml, incubated for 24h in the presence of different concentrations of the studied compounds and counted. IC_50_ values were determined and plotted here for each compound. (b) CC_50_ measured on THP-1 derived macrophages: The cells were seeded in 96-well plates (50,000 cells/well), treated with serial concentrations of each compound for 24h, and counted. CC_50_ values were determined and plotted. (c) IC_50_ measured on intracellular amastigotes: THP-1 derived macrophages were infected with *L. infantum* strain at MOI of 10:1 for 24h. Upon cell washing to eliminate residual extracellular parasites, they were further incubated for 24h in presence of different compound concentrations. Number of intracellular amastigotes and infected cells were counted after Giemsa staining and IC_50_ values were determined and plotted. (d) Effect of the compounds on *L. infantum* infected cells at a concentration of 3 *μ*M: The panel illustrates the percentage of infected THP-1 cells (i), the number of parasites per infected THP-1 (ii), and the infection index (iii). All results are shown as the mean ± SD of three independent experiments also done in technical triplicates. Statistical differences were analyzed with Student’s t-test ((* p < 0.05) or (** p < 0.01)).

In order to confirm that LieIF inhibitors also had an effect on the parasite viability, we assessed the effect of compound **208** and its 10 analogues on the viability of *L. infantum* promastigotes at the stationary phase using an MTT assay after a 24h exposure. Only the three hits, already identified as inhibitors of the ATPase activity of LieIF, affected the promastigote viability in a dose-dependent manner (S8 Fig). IC_50_ values of 4.1 *μ*M, 3.6 *μ*M and 39.1 *μ*M were obtained for compounds **208**, **20** and **48**, respectively (Fig 6(a)). The remaining eight compounds tested at different concentrations within the range of 0-100 *μ*M did not show inhibitory effects on parasite viability. The results observed at 100 *μ*M with these compounds are reported (S2 Table).

The next step was to assess the effect of different concentrations of the three compounds on macrophages derived from THP-1 cells by PMA activation, as commonly used for drug testing, [97] by using an MTT assay. All three compounds showed no significant toxicity on the THP-1 macrophages (S8 Fig). The viability of the macrophages treated with each compound was around 90–100% at the concentration corresponding to the IC_50_ on the promastigotes (S8 Fig). The CC_50_ values were determined by interpolation as 43.4 *μ*M, 35.2 *μ*M and 81.4 *μ*M for **208**, **20** and **48**, respectively. A positive correlation was observed between these CC_50_ values and the IC_50_ values obtained for the promastigotes viability. Their selectivity was thus illustrated by selectivity indexes that varied accordingly from 10.6 to 2.1 (Table 3). These selectivity values illustrated different effects on the parasite and host cell. So, we further evaluated the effect of the three compounds on the intracellular amastigote forms of *L. infantum* parasites. Upon *L. infantum* infection, THP-1 cell-derived macrophages were incubated in the presence of different compound concentrations (1 *μ*M, 3 *μ*M and 5 *μ*M for **20** and **208**; 3 *μ*M, 5 *μ*M and 25 *μ*M for compound **48**) for an additional 24h. The number of intracellular amastigotes and infected cells were then counted. An infection index was also calculated that integrated both parameters (Fig 6(d)). Untreated control cells harbored a rate of 65.1% of infected cells and a mean amastigote number per cell of 5.5, which corresponds to an infection index of 355.6. In the presence of the compounds, the number of intracellular amastigotes drastically decreased upon the 24h exposure at the different concentrations tested, in a dose-dependent manner. All three compounds also had an effect on the number of infected cells in a dose-dependent manner (S3 Table), which thus reflected on the infection index. There was also a positive correlation between the numbers of infected cells and intracellular amastigotes. Based on the amastigote numbers, we determined the IC_50_ values of the three hits (0.9–4.2 *μ*M, Table 3), which also were positively correlated to the values measured for the promastigotes and in the toxicity assays. Thus, selectivity indexes (SI) measured for the amastigotes were 31.7, 37.5 and 19.3 for compound **208**, **20** and **48**, respectively. Based on all results, the 6-*α*-aminocholestanol appeared as the more potent hit notably with a selectivity index of 37.5.

**Table 3:** Summary of the experimental results of the ATPase assays and the *in vitro* effects on the viability of the promastigotes, THP-1 cells and intramacrophage amastigotes of the three hits. SI is the ratio between the CC_50_ value against THP-1 cells and the IC_50_ value against *Leishmania* parasites (promastigotes or amastigotes, accordingly).

	LieIF	eIF4AI_Mus_	Promastigotes		Amastigotes		THP-1
	IC_50_ (*μ*M)	IC_50_ (*μ*M)	IC_50_ (*μ*M)	SI	IC_50_ (*μ*M)	SI	CC_50_ (*μ*M)
**208**	150 ± 15	115 ± 25	4.1 ± 2.6	10.6	1.4 ± 0.1	31.7	43.4 ± 0.1
**20**	160 ± 25	185 ± 25	3.6 ± 0.1	9.7	0.9 ± 0.0	37.5	35.2 ± 1.3
**48**	>1000	>1000	39.1 ± 0.8	2.1	4.2 ± 0.1	19.3	81.4 ± 2.4

## Discussion

The identification of novel drugs or targets constitutes a research priority for the treatment of leishmaniases. [7] Different criteria need to be fulfilled in order to validate a target including its absence in the host cell or the occurrence of substantial differences between the host and *Leishmania* proteins, its essentiality (demonstrated genetically or chemically), its expression in relevant stages (amastigotes in case of *Leishmania*), the presence of small molecules binding cavities, and its assayability for high throughput screening assays. [8, 98, 99] By these criteria, a list of relevant enzymes involved in metabolism, pathways or other cellular mechanisms have received attention, ranging from parasite-specific proteins to highly conserved proteins that have unique structural features impacting the protein function. [8, 95] One such example is the elongation factor 1-*α*, which was shown to be a relevant target despite its 82% identity with the mammalian orthologue. [95] Inhibitors selective to the *Leishmania* protein could be identified that targeted a structural feature unique to the parasite protein. [95] This also demonstrated the feasibility of targeting highly conserved proteins and the relevance of using virtual screenings as a cost effective approach in identifying novel inhibitors and leishmanicidal molecules.

The *L. infantum* translation initiation factor eIF4A (LieIF) was selected in this study by taking into account a range of evidence that hypothesized that it could be a novel candidate target. The study aimed at selecting inhibitors of this protein that subsequently affect *Leishmania* parasites viability by using a virtual screening process for the identification of compounds interacting with the protein combined with biochemical screening and with the biological characterization of the inhibitors. Computational approaches have the advantage of reducing the number of compounds to be screened in *in vitro* assays and thereby the costs of chemicals and the global screening procedure. Screening methods depend on the targets and pockets, the compound library and docking method used and on the strategy for effective selection of docked compounds. Of the 305 compounds selected by the strategy adopted here, four inhibitory signals were detected, corresponding to structurally different molecules. Only one compound was further characterized and confirmed as an ATPase inhibitor of LieIF and eIF4AI_Mus_, and was used as a basis for identifying additional active analogues. This original description of LieIF inhibitor series brings further evidence on the druggability of eIF4A proteins. [49]

EIF4A is the prototype of the DEAD box protein family, where members present a characteristic structural fold with the occurrence of 11 conserved motifs involved in the biochemical activity of these proteins. [16, 33] There is 50-53% identity across yeast, human and *Leishmania* eIF4As. Availability of a range of crystal structures of human and yeast eIF4A and other DEAD box proteins in the presence or absence of different ligands, [34, 37, 38, 40] facilitated comparative modeling of the *Leishmania* protein. The structure models of LieIF, presented herein, had the characteristic dumbbell shape in both bound and unbound states. Noticeably, the presence of druggable pockets in the NH_2_-terminus domain, identified as specific to LieIF *versus* the human eIF4AI, pointed to the relevance of primary sequence diversity. Biochemical analysis of LieIF highlighted significant differences in reactions’ requirements and substrate affinities between LieIF and yeast protein. [9] These differences extend to the mammalian eIF4A, as confirmed here, and infer different enzymatic properties of the eIF4A orthologues. The results obtained with the three compounds (**208**, **20** and **48**) also indicated different kinetic properties between LieIF and eIF4AI_Mus_. In fact, LieIF does not complement for the loss of yeast eIF4A in spite of its ability to bind *in vitro* to yeast eIF4G, the molecular scaffold of the eIF4F complex. In contrast, it does exert a dominant-negative phenotype in yeast resulting in growth reduction, indicating a non-productive interaction with the translation machinery. Importantly, deletion of the 25 NH_2_ residues of LieIF abolishes the dominant-negative phenotype and yields normal growth, yet without allowing complementation. This suggests significantly different molecular mechanisms and interactions across species. [9] In line with such observations, sequence divergence across species is more important in the NH_2_-terminal part of the protein including a *Leishmania*-specific insertion within a poorly conserved region (Fig 1). [9] This insertion is included in the P2 pocket, the putative RNA binding site against which we selected the compound **208**.

LieIF has interdependent ATPase and RNA helicase activities. Notably, we confirmed its assayability and established a simple RNA-dependent ATPase assay that uses the malachite green to measure the amount of Pi released. [9] Herein, it was adapted to fit 96-well microtiter plates, and statistical evaluation provided robust Z’-scores (> 0.5) indicating that the assay is reliable. The screening assay used 500 *μ*M compound concentrations, justified by the high protein amount engaged (1 *μ*M) as eIF4A activities *in vitro* are poor. [9, 100] Under these conditions, compound **208** selected against the P2 pocket showed efficient ATPase inhibition (90%), and presented IC_50_ values lower than the K_m_ value for ATP. Actually, IC_50_ measures depend on reaction conditions notably the amount of protein (1 *μ*M) and substrate (1 mM) engaged in the reaction, so here it corresponds to 150-fold excess over the protein. To our surprise, the compound also reacted with eIF4AI_Mus_ with a comparable IC_50_ value (115 ± 25 *μ*M), but with different kinetic properties. One explanation could be the occurrence of a different binding site on the mammalian eIF4AI, as supported by the modeling and docking results. The present work leaves open questions on the inhibition mode and the interaction of the compound with the proteins. Work is in progress to address these questions.

To ascertain the interest of **208**, 10 structurally related analogues were selected and tested on both proteins. Two compounds, **20** and **48**, inhibited eIF4AI_Mus_ and LieIF with different efficiencies. None of the other eight compounds were shown to be active against the two proteins. Similar IC_50_ values were obtained for each protein but as seen with **208** the kinetic properties were different according to the protein. Importantly, none of the eight inactive compounds had an effect on *Leishmania* promastigotes, the extracellular form of the parasite. However, all ATPase inhibitors (**208**, **20** and **48**) negatively impacted viability of the promastigotes with low IC_50_ values (4.1, 3.6 and 39.1 *μ*M, respectively) that were positively correlated with those determined for LieIF ATPase assays (150, 160 *μ*M and > 1mM, respectively). The three compounds presented CC_50_ values on macrophage cells that reflected the potency of the ATPase inhibitors but at a higher concentration range than on the parasite (43.4, 35.2 and 81.4 *μ*M, respectively), indicating a more potent effect on the parasite than on the host cells. These compounds also similarly reacted on the intracellular amastigotes (1.4, 0.9 and 4.2 *μ*M respectively) and demonstrated even better selectivity indexes (37.5–19.3) than with the promastigotes (10.6–2.1) as expected in drug screening campaigns. [101] The difference between the IC_50_ values on the enzymes (150 - > 1000 *μ*M) and those on the parasite (< 1–40 *μ*M) could be explained by the fact that the amounts of protein used in the assay are far above physiological concentrations determined in *Leishmania*. [19] In addition, the activity is measured on the proteins as single units but eIF4A is a member of a multimeric complex. Notably, it is well known that the activity of eIF4A is enhanced by cofactors, and it can reach 20-fold increase upon association with its partner proteins, such as the components of the pre-initiation complex [102–104] or even under molecular crowding. [105] In addition, the study did not investigate effects on the RNA helicase, the other enzymatic activity ensured by these proteins. So, the effect of these compounds could be more pronounced or more selective on this activity.

Our hits consisted of amine and ketone cholestanol scaffolds. Compound **208** is an epimeric mixture of 6-*α*/*β*-aminocholestanol. Compound **20** is the *α*-epimer and compound **48** is the 6-ketocholestanol. Far less effective, compound **48** presents a ketone group replacing the amino group on carbon C6 on **208** and **20** (Fig 4(a)). The amino group is also absent on the eight analogues inactive on the ATPase activity. Even the addition of reactive chemical groups at the same position could not ensure activity (S6 Fig). This indicates the importance of the amino group in the protein-inhibitor interactions. Docking of the three hits on P2 supported this hypothesis, and it is in line with experimental results predicting better and more efficient interactions of the 6-*α*-aminocholestanol with P2 residues, as compared to the *β*-epimer or the 6-ketocholestanol. These aminocholestanols were described as anti-fungal molecules that reduced yeast growth at low micromolar concentrations (∼ 31 *μ*M), presumably by targeting ergosterol synthesis. [96, 106] Sterol derivatives that interfere with ergosterol biosynthesis, and presenting a chemical relatedness to our hits, were described for their leishmanicidal activities. [107] Noticeably, sterol derivatives such as the 7-*α*/*β*-aminocholesterol reduced by 59% the number of intracellular *L. donovani* (another VL agent) amastigotes at 1.94 *μ*M concentration, but it demonstrated a low selectivity index (∼ 3). [107] Its structure presents a double bond on the second ring, which confers a local planar 3D shape to this molecule, compared to our hits, in addition to a displacement of the amino group on carbon C7. This study also hypothesized that this aminocholesterol could target ergosterols biosynthesis, but with no experimental evidence provided. [107] Our results do not permit us to exclude interactions with other targets, but there is clear biochemical evidence for the interaction of the cholestanol-based inhibitors (virtually selected without prior reference to literature) with LieIF, and there is a positive correlation between the potencies of enzyme inhibition and leishmanicidal effects of the three molecules. These compounds also bear a distant similarity to hippuristanol, a selective inhibitor of the mammalian eIF4A, [108] thought to act as an allosteric inhibitor of RNA binding in the C-terminal domain of eIF4A. [108–110] It inhibits eIF4A helicase activity by blocking the protein in the closed conformation, [111] and it is unable to affect the activity of other DBPs like human DDX19 and DDX52. [108] No evidence is available on its effect on LieIF or on *Leishmania*. This or other eIF4A inhibitors will need to be tested on LieIF and their cidal effects assessed on *Leishmania*.

The role of eIF4A proteins is pivotal as an essential enzyme of the eIF4F translation initiation complex. [17, 18] Its essentiality has been genetically confirmed in yeast, [31] mammals [112] and in *Trypanosoma brucei*, another kinetoplastid parasite. [19] However, as RNAi is not applicable in the *Leishmania* subgenus, and the gene is organized as a cluster of two identical tandem copies on the likely polyploid chromosome 1, [9, 113] genetic confirmation of its essentiality is difficult. With the advent of CRISP-Cas9 technology, strategies may be deployed to confirm the essentiality of LieIF in *L. infantum* [114, 115] and to assess the biological relevance of the interactions of the inhibitors with LieIF as has been shown for rocaglates and eIF4AI. [116]

This study constitutes a first step towards validation of LieIF as a drug target. It delivers novel eIF4A inhibitors. As shown here, the 6-*α*-aminocholestanol with IC_50_ value lower than 1 *μ*M on intracellular amastigotes, little toxicity and a selectivity index higher than 20, constitutes a promising anti-*Leishmania* molecule that deserves further investigation.

## Supporting information

S1 Table Summary of Ramachandran plots for LieIF models obtained by Modeller. For each model, the percentage of residues present within the favored, the allowed and the outlier regions of the plot are reported. The structures selected as apo-LieIF and holo-LieIF are in bold.
(PDF)

S2 Table Test of compounds on promastigotes viability with MTT assay. Effect of the identified analogues on *L. infantum* promastigotes tested at 100 *μ*M. The results represent the mean ± SD of three independent experiments. Results with compounds R209988 (g) and R210137 (i) were unstable and thus are not reported. Compound numbers’ as in figure S6 Fig are shown in brackets.
(PDF)

S3 Table *In vitro* anti-leishmanial activity of the three hits against intramacrophage amastigotes of *L. infantum* LV50. IPI = 100 − (Inhibition Index in treated cells/Inhibition Index in untreated cells * 100).
(PDF)

S1 Fig Ramachandran plots for LieIF models. (a) Holo-LieIF presented 8 residues in the allowed region and 2 in the outlier. (b) Apo-LieIF presented 12 residues in the allowed region and 7 in the outlier. (c) Apo-LieIF_trunc/MD_ presented 26 residues in the allowed region and 3 in the outlier.
(PDF)

S2 Fig RMSD and B-factor variations for apo-LieIF (in black), holo-LieIF (in red) and mammalian eIF4AI (chain A of the PDB entry: 3EIQ) trajectories. (a) RMSD variation during 2ns trajectories. (b) B-factor fluctuation for each residue of the truncated structures of LieIF [AA 25-396].
(PDF)

S3 Fig Cavities detected using *mkgrid* on the 2ns MD trajectory of apo-LieIF_trunc/MD_, holo-LieIF_trunc/MD_ and on the mammalian orthologue eIF4AI (PDBid = 3EIQ_A). Panels (a), (c) and (e) show all detected cavities in colored mesh grid and a cartoon representation of the proteins. Panel (b) shows pockets P1 (in orange) and P2 (in blue) identified on apo-LieIF_trunc/MD_. Panel (d) shows holo-LieIF_trunc/MD_ with a cavity that appears on an equivalent location to P2 (showed by a star), located on the protein surface. All other cavities were either located on the surface or presented small volumes (≤ 100 Å^3^), except for the inter-domain cleft. Thus, no cavities detected on holo-LieIF_trunc/MD_ were retained for the virtual screening. Panel (f) shows the human eIF4AI with no equivalent pockets to P1 or P2.
(PDF)

S4 Fig SOMs obtained on VS results. (a) uMatrix corresponding to the SOM obtained for Dock results targeting P1. (b) Dock scores projected on the SOM shown in (a). (c) uMatrix corresponding to the SOM obtained for Dock results targeting P2. (d) Dock scores projected on the SOM represented in (c). (e) uMatrix corresponding to the SOM obtained for ADvina results targeting P2. (f) ADvina scores projected on the SOM represented in (e).
(PDF)

S5 Fig Histograms of docking scores distributions obtained with Dock on the non-phophorylated form of pocket P2 (in blue) and on the phosphorylated form of P2 (in green). A shift to positive scores was observed when docking on the phosphorylated form of P2, indicating a relevant effect of the phosphorylated THR135 on the protein-ligand interactions.
(PDF)

S6 Fig Chemical structures of the selected analogues of compound 208. (a) Compound **20** like **208** was obtained from the chemists at the Université de Caen de Basse-Normandie, Centre d’Études et de Recherche sur le Médicament de Normandie (CERMN), UFR des Sciences Pharmaceutiques. (b-j) The remaining nine compounds were purchased from Sigma Aldrich. Their identifiers are shown below the corresponding structures.
(PDF)

S7 Fig Docking poses of all three hits on pocket P2 on apo-LieIF_trunc/MD_. (a) Best docking pose of 6-*α*-aminocholestanol (**20**) (b) Best docking pose of 6-*β*-aminocholestanol (*β*-epimer of **208**) (c) Best docking pose of 6-ketocholestanol (**48**).
(PDF)

S8 Fig MTT cell viability assay showing a promising anti-leishmanial activity of compounds 208, 20 and 48 in a dose-dependent manner, and a little cellular toxicity at the active concentrations. (a) Effect of the identified novel inhibitors on *L. infantum* promastigotes. (b) Effect of the identified novel inhibitors on THP-1-derived macrophages.
(PNG)
