# Supplementary material for: Identification of novel leishmanicidal molecules by virtual and biochemical screenings targeting Leishmania eukaryotic translation initiation factor 4A
Source: PLoS Negl Trop Dis. 2018 Jan 18;12(1):e0006160. doi: 10.1371/journal.pntd.0006160 (PMC5790279; doi:10.1371/journal.pntd.0006160)
Supplement: S1 Table — For each model, the percentage of residues present within the favored, the allowed and the outlier regions of the plot are reported. The structures selected as apo-LieIF and holo-LieIF are in bold. (PDF) [file pntd.0006160.s001.pdf]

Ligand-free model N°	01	02	03	**04**	05	06	07	08	09	10
Favored (98%)	93.0%	95.3%	94.0%	**95.3%**	94.5%	94.0%	94.3%	93.8%	91.3%	91.8%
Allowed (2%)	4.2%	3.0%	4.5%	**3.0%**	3.2%	3.5%	4.0%	4.5%	6.2%	5.7%
Outlier (0%)	2.7%	1.7%	1.5%	**1.7%**	2.2%	2.5%	1.7%	1.7%	2.5%	2.5%

Substrate-bound model N°	01	02	**03**	04	05	06	07	08	09	10
Favored (98%)	97.5%	96.8%	**97.5%**	97.3%	97.3%	97.3%	95.5%	96.0%	97.3%	96.5%
Allowed (2%)	1.7%	2.5%	**2.0%**	2.0%	2.2%	2.0%	3.5%	3.0%	2.2%	3.0%
Outlier (0%)	0.7%	0.7%	**0.5%**	0.7%	0.5%	0.7%	1.0%	1.0%	0.5%	0.5%
