# Supplementary material for: Identification of novel leishmanicidal molecules by virtual and biochemical screenings targeting Leishmania eukaryotic translation initiation factor 4A
Source: PLoS Negl Trop Dis. 2018 Jan 18;12(1):e0006160. doi: 10.1371/journal.pntd.0006160 (PMC5790279; doi:10.1371/journal.pntd.0006160)
Supplement: S2 Table — Effect of the identified analogues on L. infantum promastigotes tested at 100 μM. The results represent the mean ± SD of three independent experiments. Results with compounds R209988 (g) and R210137 (i) were unstable and thus are not reported. Compound numbers’ as in figure S6 Fig are shown in brackets. (PDF) [file pntd.0006160.s002.pdf]

Compound reference	LV50 promastigotes viability (%)
R205761 (b)	120 ± 24
R206644 (c)	80 ± 24
R210552 (d)	116 ± 11
R222283 (e)	106 ± 6
R205435 (f)	113 ± 6
R209988 (g)	–
R210137 (i)	–
R184314 (j)	60 ± 6
