# Supplementary material for: Identification of novel leishmanicidal molecules by virtual and biochemical screenings targeting Leishmania eukaryotic translation initiation factor 4A
Source: PLoS Negl Trop Dis. 2018 Jan 18;12(1):e0006160. doi: 10.1371/journal.pntd.0006160 (PMC5790279; doi:10.1371/journal.pntd.0006160)
Supplement: S3 Table — IPI = 100 − (Inhibition Index in treated cells/Inhibition Index in untreated cells * 100). (PDF) [file pntd.0006160.s003.pdf]

Compounds	Concentration (μM)	(μg/ml)	% infected cells	Number of amastigotes/cell	Infection Index	Inhibition Parasite Index
**208**	5	2.02	26.50 ± 9.2	0.79 ± 0.0	20.93 ± 8.6	94.11 ± 6.1
	3	1.21	27.00 ± 7.9	0.99 ± 0.3	26.73 ± 14.1	92.48 ± 5.6
	1	0.40	40.00 ± 2.8	3.15 ± 0.0	126.00 ± 7.9	64.56 ± 4.7
**20**	5	2.02	34.50 ± 9.2	1.74 ± 1.1	60.03 ± 55.5	83.11 ± 8.6
	3	1.21	37.00 ± 1.4	2.06 ± 0.3	76.22 ± 13.1	78.56 ± 4.6
	1	0.40	41.50 ± 2.1	2.48 ± 0.0	102.92 ± 6.1	71.05 ± 0.8
**48**	25	10.07	21.00 ± 1.4	0.90 ± 0.2	18.90 ± 6.2	94.68 ± 1.7
	5	2.01	43.00 ± 7.1	2.34 ± 0.5	100.62 ± 20.5	71.70 ± 13.4
	3	1.21	41.50 ± 2.1	3.32 ± 0.0	137.78 ± 7.9	61.24 ± 4.7
CNTRL	-	-	65.11 ± 4.5	5.46 ± 1.0	355.50 ± 145.3	-
