# Supplementary figures and images for: Identification of novel leishmanicidal molecules by virtual and biochemical screenings targeting Leishmania eukaryotic translation initiation factor 4A
Source: PLoS Negl Trop Dis. 2018 Jan 18;12(1):e0006160. doi: 10.1371/journal.pntd.0006160 (PMC5790279; doi:10.1371/journal.pntd.0006160)

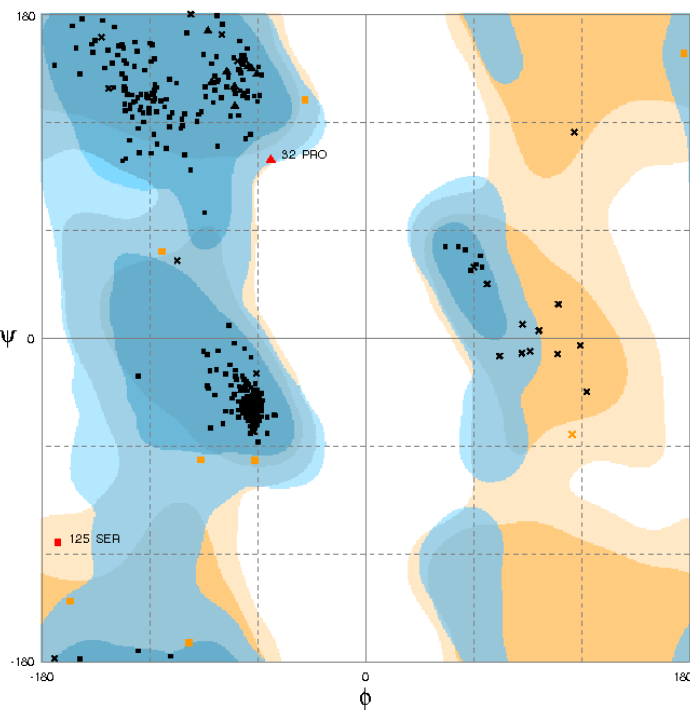

(a) Holo-LieIF

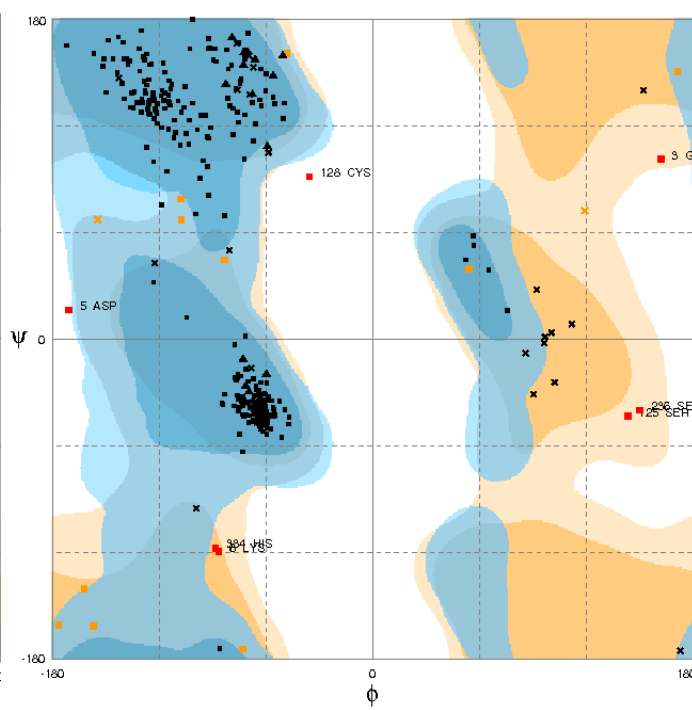

(b) Apo-LieIF

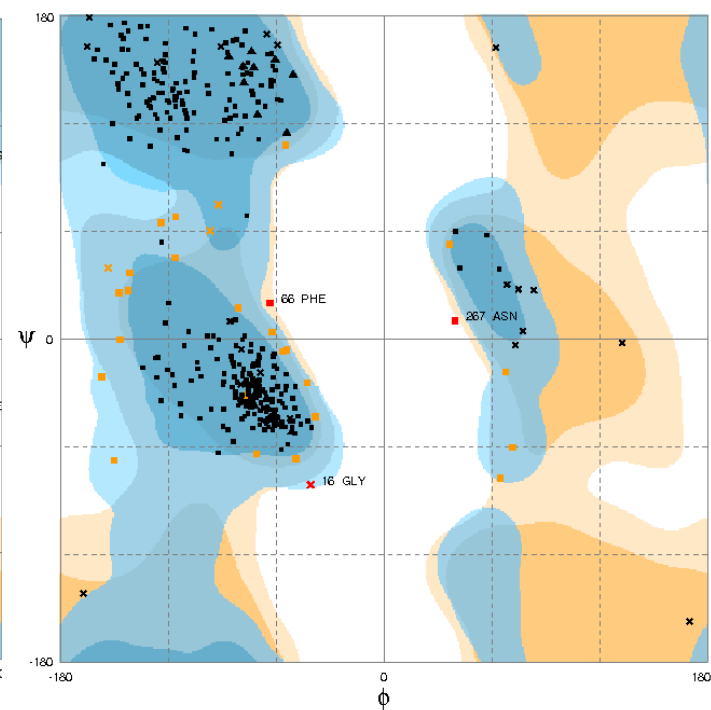

(c) Apo-LieIF<sub>trunc/MD</sub>

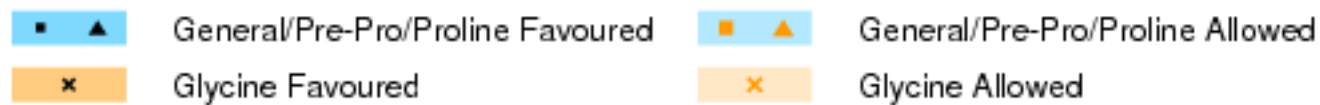

Supplement: S1 Fig — (a) Holo-LieIF presented 8 residues in the allowed region and 2 in the outlier. (b) Apo-LieIF presented 12 residues in the allowed region and 7 in the outlier. (c) Apo-LieIFtrunc/MD presented 26 residues in the allowed region and 3 in the outlier. (PDF) [file pntd.0006160.s004.pdf]

**(a)**

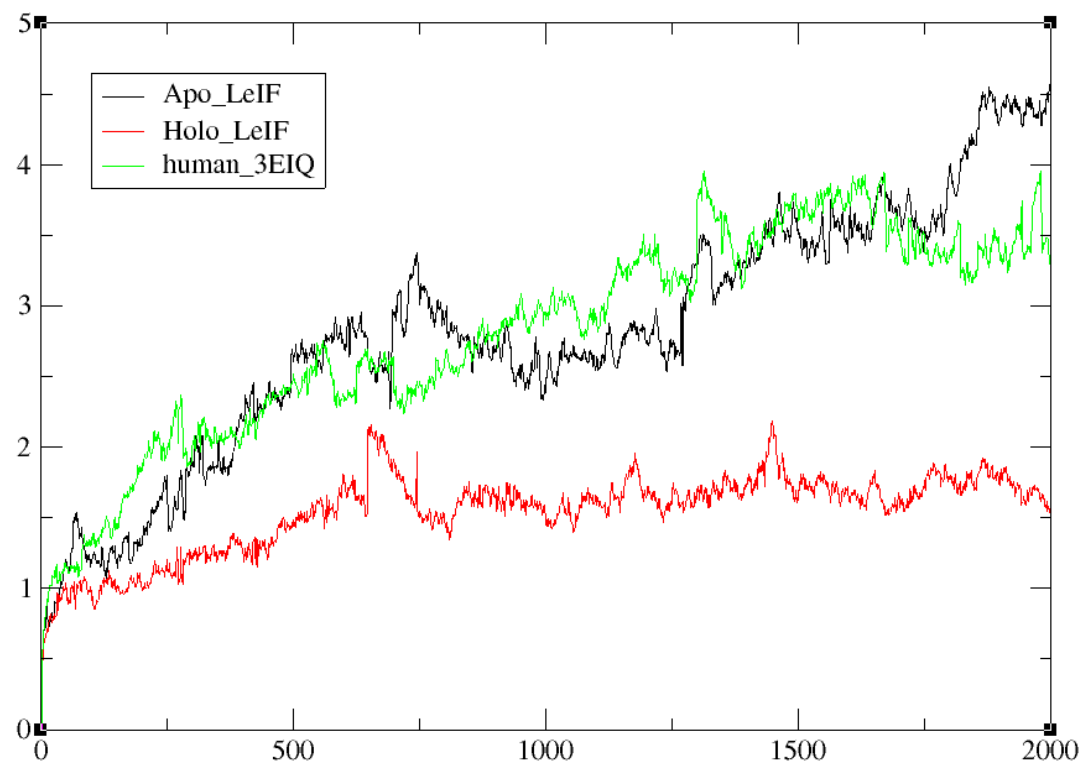

**(b)**

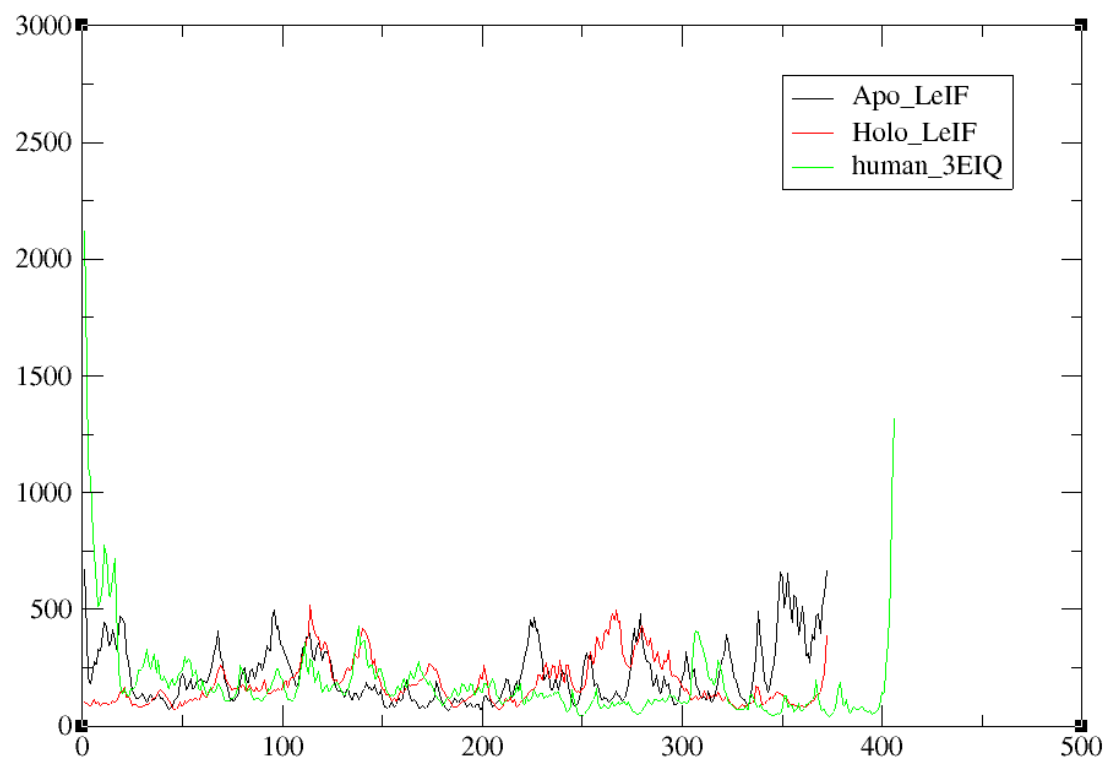

Supplement: S2 Fig — (a) RMSD variation during 2ns trajectories. (b) B-factor fluctuation for each residue of the truncated structures of LieIF [AA 25-396]. (PDF) [file pntd.0006160.s005.pdf]

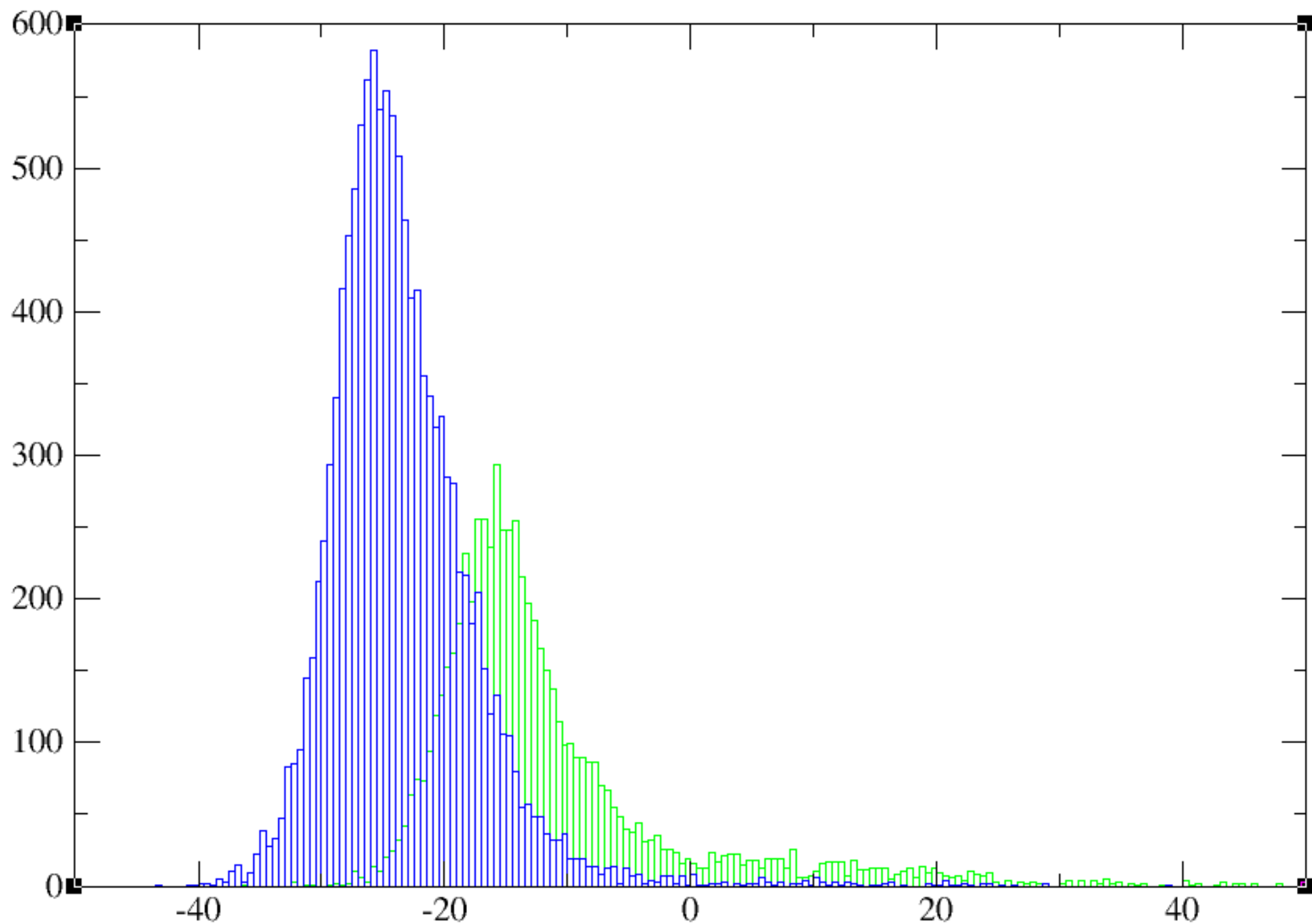

Dock scores on pocket P2

Supplement: S5 Fig — A shift to positive scores was observed when docking on the phosphorylated form of P2, indicating a relevant effect of the phosphorylated THR135 on the protein-ligand interactions. (PDF) [file pntd.0006160.s008.pdf]

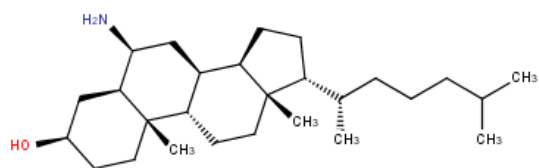

(a) MR26620 (**20**)

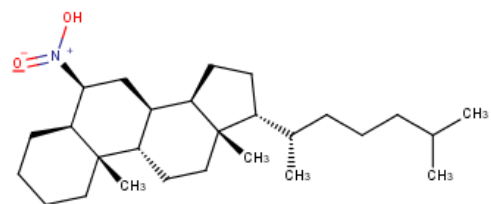

(b) R205761

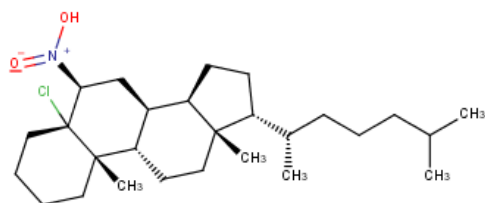

(c) R206644

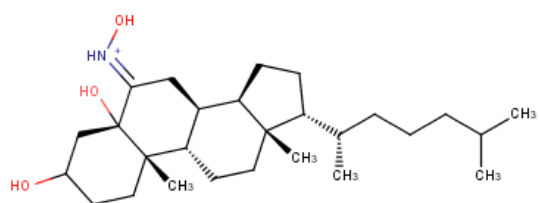

(d) R210552

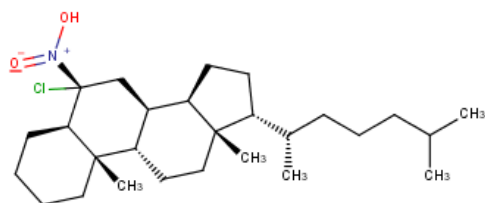

(e) R222283

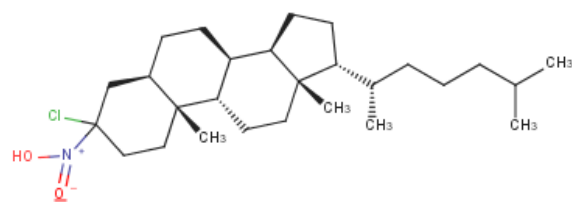

(f) 205435

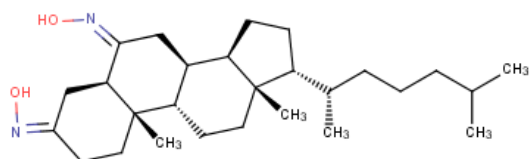

(g) R209988

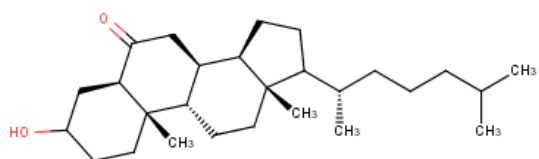

(h) K1250 (**48**)

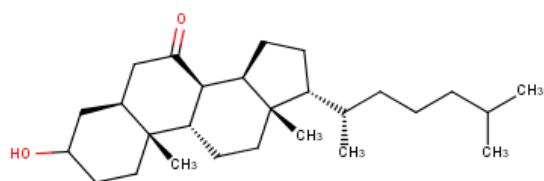

(i) R210137

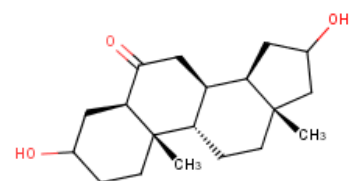

(j) R184314

Supplement: S6 Fig — (a) Compound 20 like 208 was obtained from the chemists at the Université de Caen de Basse-Normandie, Centre d’Études et de Recherche sur le Médicament de Normandie (CERMN), UFR des Sciences Pharmaceutiques. (b-j) The remaining nine compounds were purchased from Sigma Aldrich. Their identifiers are shown below the corresponding structures. (PDF) [file pntd.0006160.s009.pdf]

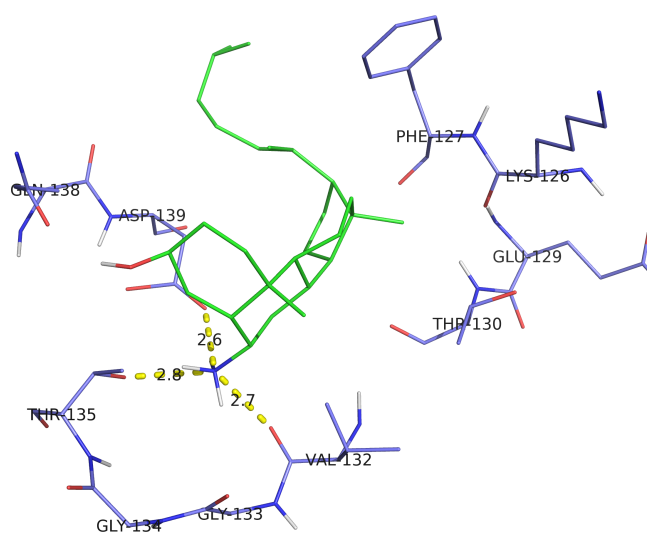

(a) 6- $\alpha$ -aminocholestanol

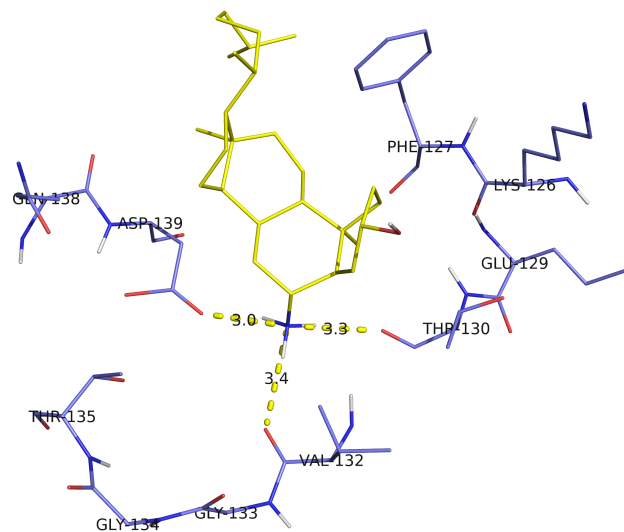

(b) 6- $\beta$ -aminocholestanol

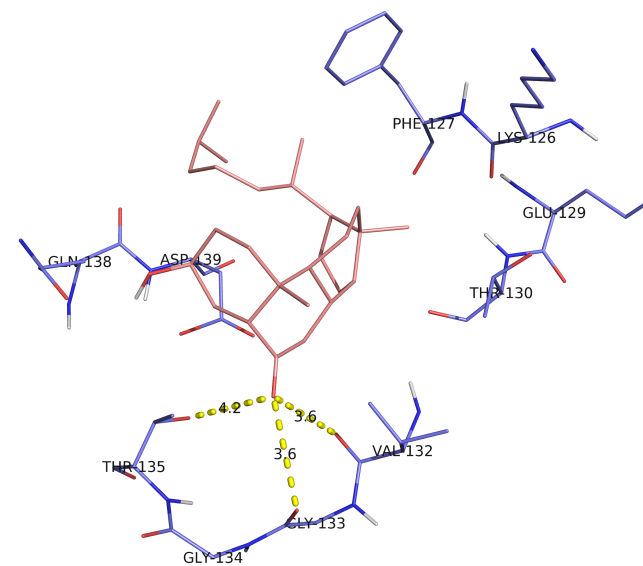

(a) 6-ketocholestanol

Supplement: S7 Fig — (a) Best docking pose of 6-α-aminocholestanol (20) (b) Best docking pose of 6-β-aminocholestanol (β-epimer of 208) (c) Best docking pose of 6-ketocholestanol (48). (PDF) [file pntd.0006160.s010.pdf]

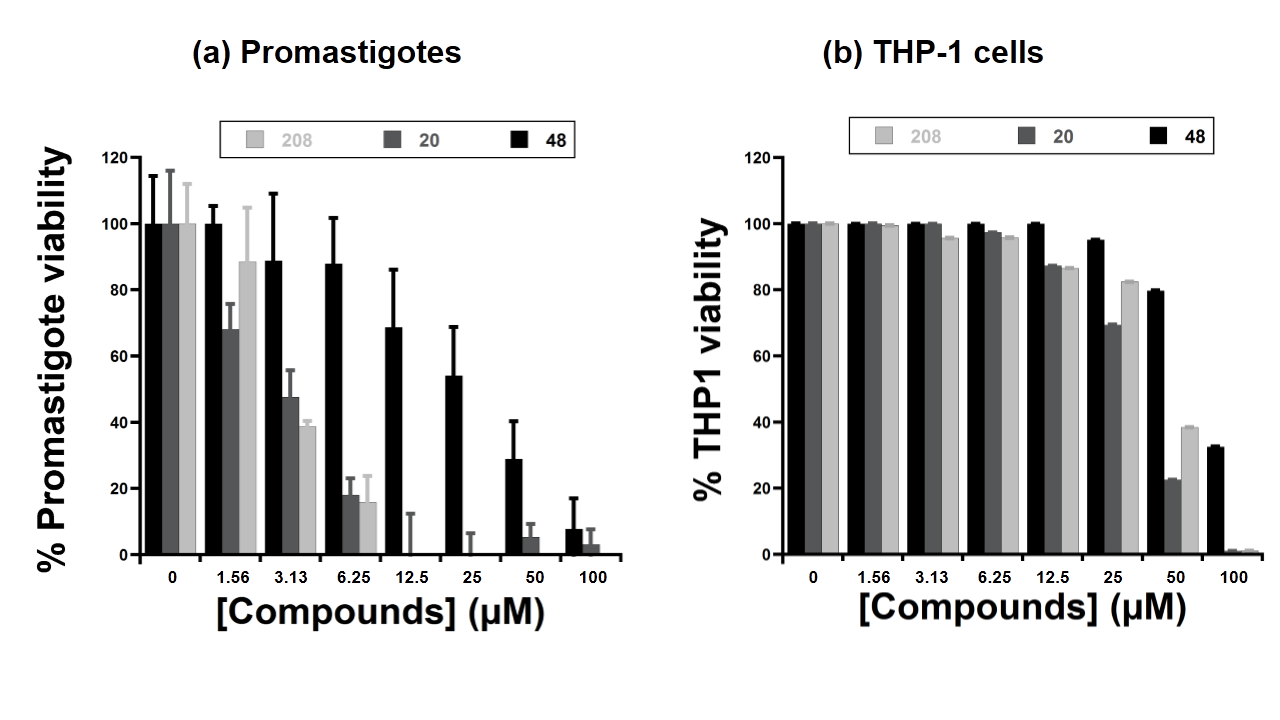

Supplement: S8 Fig — (a) Effect of the identified novel inhibitors on L. infantum promastigotes. (b) Effect of the identified novel inhibitors on THP-1-derived macrophages. (PNG) [file pntd.0006160.s011.png]
