# Supplementary material for: Identification of novel leishmanicidal molecules by virtual and biochemical screenings targeting Leishmania eukaryotic translation initiation factor 4A
Source: PLoS Negl Trop Dis. 2018 Jan 18;12(1):e0006160. doi: 10.1371/journal.pntd.0006160 (PMC5790279; doi:10.1371/journal.pntd.0006160)
Supplement: S3 Fig — Panels (a), (c) and (e) show all detected cavities in colored mesh grid and a cartoon representation of the proteins. Panel (b) shows pockets P1 (in orange) and P2 (in blue) identified on apo-LieIFtrunc/MD. Panel (d) shows holo-LieIFtrunc/MD with a cavity that appears on an equivalent location to P2 (showed by a star), located on the protein surface. All other cavities were either located on the surface or presented small volumes (≤ 100 Å3), except for the inter-domain cleft. Thus, no cavities detected on holo-LieIFtrunc/MD were retained for the virtual screening. Panel (f) shows the human eIF4AI with no equivalent pockets to P1 or P2. (PDF) [file pntd.0006160.s006.pdf]

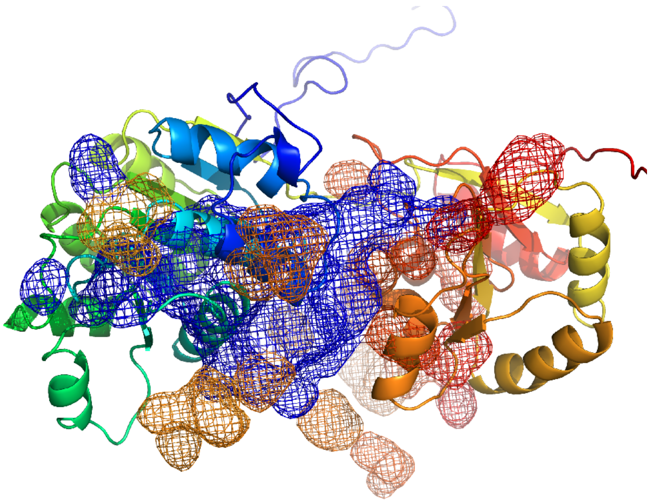

(a) Apo-LieIF$_{\mathrm{trunc/MD}}$

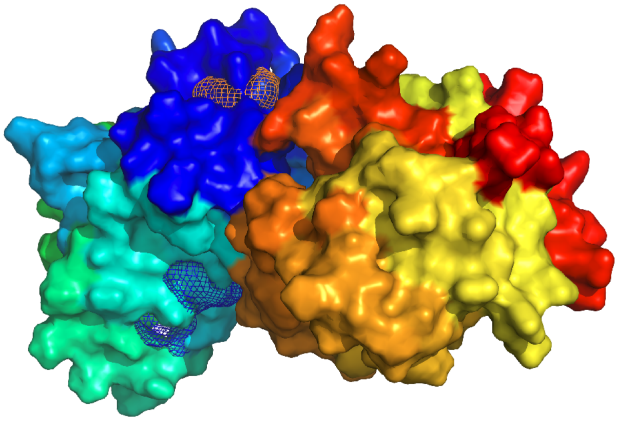

(b) Apo-LieIF$_{\mathrm{trunc/MD}}$

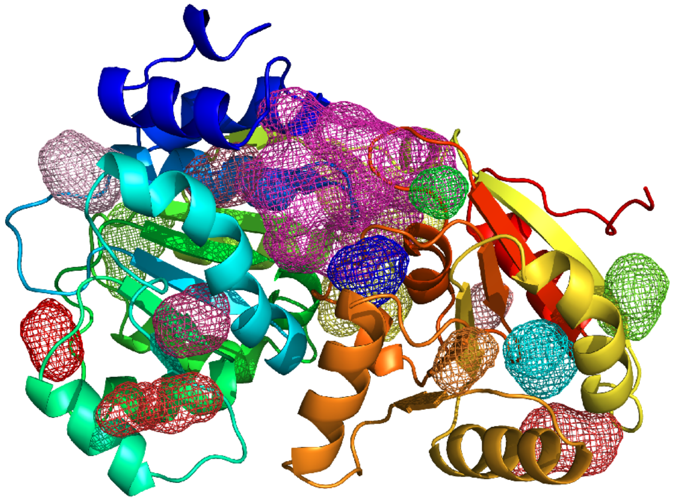

(c) Holo-LieIF$_{\mathrm{trunc/MD}}$

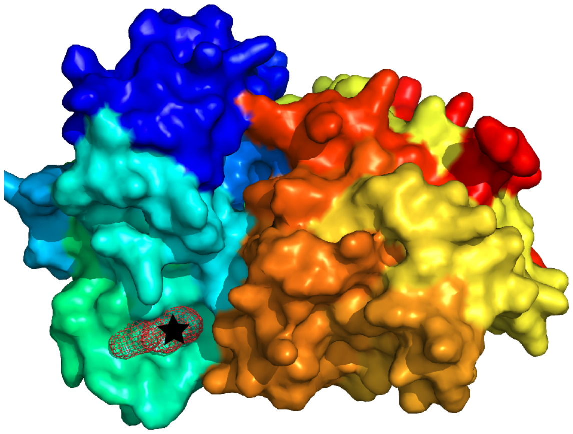

(d) Holo-LieIF$_{\mathrm{trunc/MD}}$

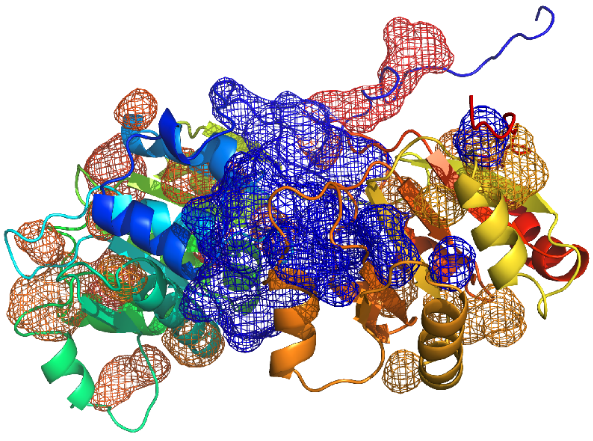

(e) Human eIF4AI

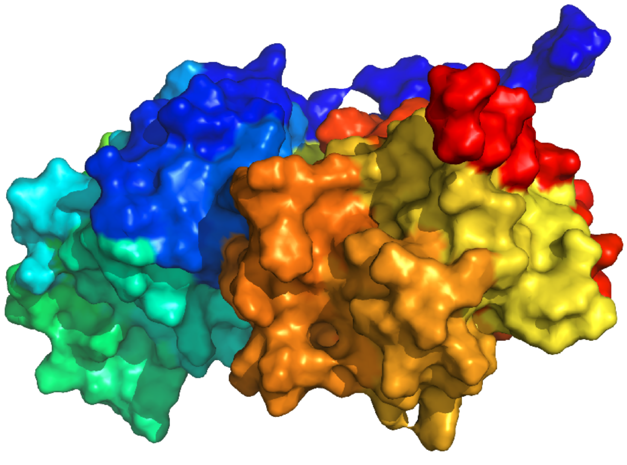

(f) Human eIF4AI
