# Supplementary material for: Identification of novel leishmanicidal molecules by virtual and biochemical screenings targeting Leishmania eukaryotic translation initiation factor 4A
Source: PLoS Negl Trop Dis. 2018 Jan 18;12(1):e0006160. doi: 10.1371/journal.pntd.0006160 (PMC5790279; doi:10.1371/journal.pntd.0006160)
Supplement: S4 Fig — (a) uMatrix corresponding to the SOM obtained for Dock results targeting P1. (b) Dock scores projected on the SOM shown in (a). (c) uMatrix corresponding to the SOM obtained for Dock results targeting P2. (d) Dock scores projected on the SOM represented in (c). (e) uMatrix corresponding to the SOM obtained for ADvina results targeting P2. (f) ADvina scores projected on the SOM represented in (e). (PDF) [file pntd.0006160.s007.pdf]

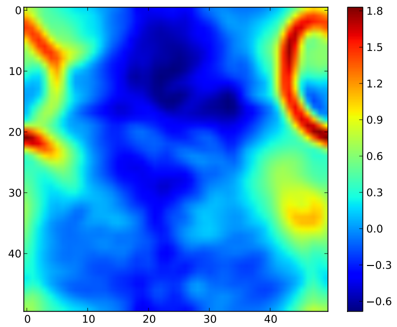

(a) uMatrix for P1 (Dock)

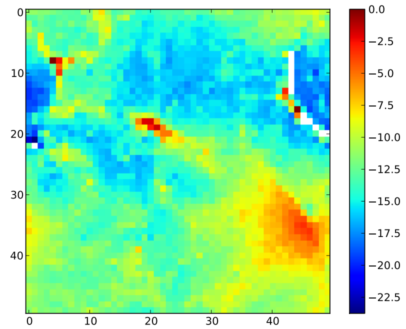

(b) Docking scores for P1 (Dock)

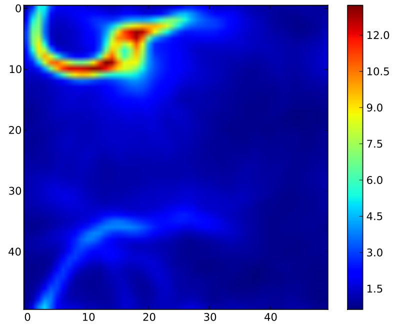

(c) uMatrix for P2 (Dock)

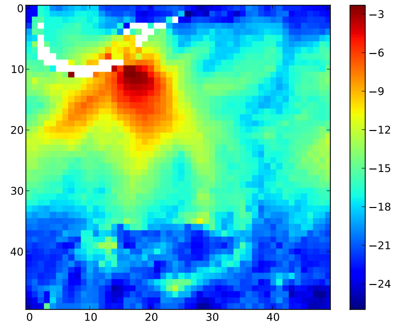

(d) Docking scores for P2 (Dock)

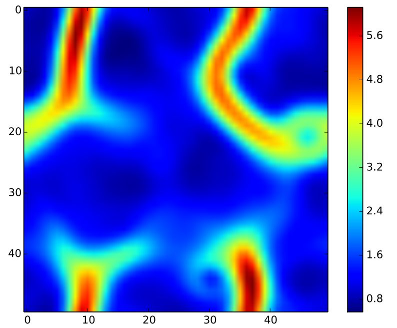

(e) uMatrix for P2 (ADvina)

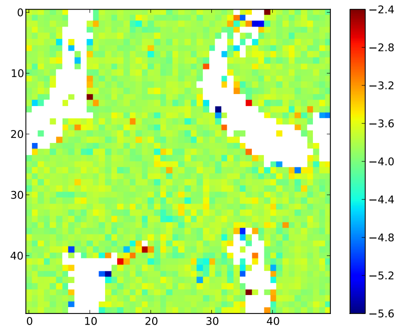

(f) Docking scores for P2 (Dvina)
